# Osteocalcin-dependent and -independent metabolic dysregulation in a mouse model of Osteogenesis imperfecta

**DOI:** 10.1038/s41413-026-00553-1

**Published:** 2026-07-16

**Authors:** Josephine T. Tauer, Frank Rauch, Mathieu Ferron, Svetlana V. Komarova

**Affiliations:** 1 Department of Pediatrics and Adolescent Medicine, Johannes Kepler University, Linz, Austria; 2 Research Centre, Shriners Hospital for Children-Canada, Montreal, QC Canada; 3 Department of Pediatrics, McGill University, Montreal, QC Canada; 4 Unité de Recherche en Physiologie Moléculaire, Institut de Recherches Cliniques de Montréal, Montréal, QC Canada; 5 Départements de Médecine et de Biochimie et Médecine Moléculaire, Université de Montréal, Montréal, QC Canada; 6 Faculty of Dental Medicine and Oral Health Sciences, McGill University, Montreal, QC Canada; 7 Department of Biomedical Engineering, Faculty of Engineering, University of Alberta, Edmonton, AB Canada

**Keywords:** Bone, Fat metabolism, Osteogenesis imperfecta

## Abstract

Osteogenesis imperfecta (OI) is a rare bone fragility disorder. Previously, in a severe OI mouse model (*Col1a1*^*Jrt/+*^), a sex- and age-dependent metabolic phenotype was observed, correlating with elevated levels of the bone-derived hormone osteocalcin (OCN). This hormone is known to play a crucial role in managing energy metabolism, including glucose regulation and fat mass. In fact, upon high-fat diet (HFD) exposure, OI mice developed a metabolic syndrome linked to sex and OCN. To assess OCN’s role in OI, *Col1a1*^*Jrt/+*^ mice were crossed with OCN-deficient mice (*Bglap*). Under regular chow and HFD conditions, both OCN-dependent and OCN-independent metabolic alterations were identified. OCN-dependent processes were adipose tissue, liver, and insulin metabolism in a sex-, age-, and diet-dependent manner. OCN-independent traits included the pancreas in juvenile mice, HFD-induced pancreatic insulin levels and glucose intolerance, besides overall growth, fertility, and bone phenotype. Notably, increased juvenile energy expenditure was OCN-independent, while HFD-induced changes were OCN-driven. These findings demonstrate OCN’s role in shaping the metabolic phenotype while revealing distinct OCN-independent effects, emphasizing the complex genetic regulation of metabolism in OI.

## Introduction

Osteogenesis imperfecta (OI) is a rare inherited skeletal disorder marked by high bone turnover and elevated circulating levels of osteocalcin (OCN).^1–3^ The abnormal bone metabolism in OI is attributed to altered bone formation^4,5^ due to the disruption of collagen type I production and assembly, resulting from mutations in the genes encoding collagen type I (*COL1A1* and *COL1A2)* or those involved in collagen processing.^6^ The clinical features of OI include bone fragility, short stature, bone deformities, alongside non-skeletal symptoms like reduced muscle mass and function, dentinogenesis imperfecta, blue sclerae, and cardio/respiratory issues.^6–8^ Notably, pediatric OI patients display a state of hypermetabolism^9–11^ that appears to lessen with age; however, adults with OI often have normal or increased fat mass,^12–14^ indicating age-related changes in metabolism.

OCN, a hormone produced by bone-forming osteoblasts, was initially thought to primarily contribute to bone mineralization. However, it has since been recognized as a bone-derived endocrine hormone that plays a critical role in regulating energy metabolism, including glucose homeostasis and fat mass^15–17^; although contradictory results have also been published.^18–20^ However, using the *Col1a1*^*Jrt/+*^ mouse model, which mimics dominant moderate-to-severe OI,^21^ we previously identified several metabolic alterations. These included increased insulin levels in males, improved glucose tolerance in females, lower levels of random glucose, low adiposity in both sexes, and increased energy expenditure.^22^ In conjunction with this, these mice displayed significantly elevated levels of total osteocalcin (tOCN), including both its carboxylated (GLA13-OCN) and uncarboxylated (GLU-OCN) forms.^22^ Moreover, when subjected to a long-term high-fat diet (HFD), *Col1a1*^*Jrt/+*^ mice developed HFD-induced metabolic syndrome in a sex-dependent manner.^23^ Female OI mice exhibited obesity, hyperglycemia, and glucose intolerance due to HFD, while their male counterparts remained protected from HFD-induced obesity. This protective effect in OI males was linked to increased activity, energy expenditure, and brown adipose tissue (BAT) thermogenesis, which correlated with elevated serum OCN levels.^23^ Consequently, we concluded that male *Col1a1*^*Jrt/+*^ mice experienced a synergistic effect from HFD- and OCN-induced BAT thermogenesis, indicating that OCN may drive the metabolic phenotype in OI. Likewise, increased energy expenditure and elevated OCN levels were noted in another severe OI mouse model, *oim/oim* mice,^24,25^ further supporting the hypothesis that OCN mediates metabolic alterations in OI.

This study aimed to investigate whether OCN drives metabolism in OI. We crossed OI mice (*Col1a1*^*Jrt/+*^ mice, FVB background) with OCN-KO mice (*Bglap*; C57BL6J background) to produce OI mice with either reduced (OI/OCN-Het) or absent OCN (OI/OCN-KO) on a mixed background. We then comprehensively characterized metabolic phenotype in male and female OI, OI/OCN-Het, and OI/OCN-KO mice on a mixed FVB/C57BL6J background fed a regular chow diet. In addition, we examined OCN effects in male and female OI and OI/OCN-KO mice fed a long-term custom low-fat (LFD) or HFD. Here, we demonstrate that the inactivation of OCN partially rescues metabolic phenotype in *Col1a1*^*Jrt/+*^ mice, suggesting that OCN-dependent and OCN-independent mechanisms contribute to the metabolic phenotype in OI.

## Results

### Generation of osteocalcin-deficient OI mice

**Fig. 1:**
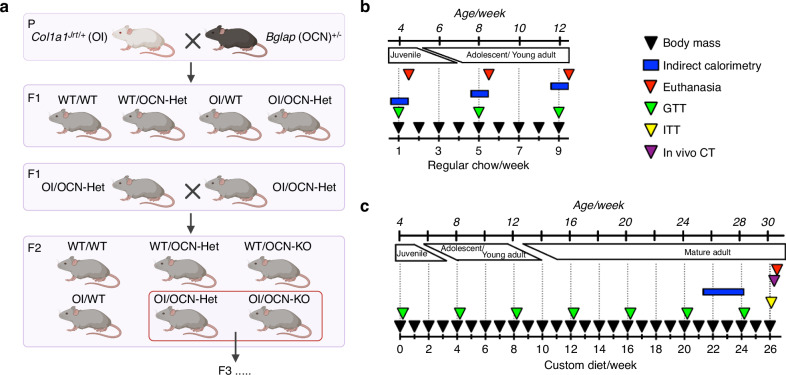
Overview of breeding strategy (**a**) and experimental design of analysis on regular chow (**b**) or long-term custom-diet intervention (**c**) in OI/OCN mice. **a** OCN-deficient *Col1a1*^*Jrt/+*^ mice (OI/OCN) were generated by first crossing heterozygous *Col1a1*^*Jrt/+*^ mice (OI) with heterozygous *Bglap*^+/-^ mice (OCN) to generate *Col1a1*^*Jrt/+*^/*Bglap*^*+/-*^ (OI/OCN-Het) and *Col1a1*^*+/+*^/*Bglap*^*+/-*^ (OCN-Het) mice. Next, OI/OCN-Het and OI/OCN-Het mice were crossed to generate *Col1a1*^*Jrt/+*^/*Bglap*^*-/-*^ mice (OI/OCN-KO). **b** Mice were maintained on regular chow from weaning at the age of 3–3.5 weeks. **c** Mice were maintained on custom diets from 4 to 30 weeks of age. **b**, **c** The following assessments were performed as indicated: body mass (black triangles, weekly), glucose tolerance test (green triangles, every 4 weeks), insulin tolerance test (yellow triangle, once), indirect calorimetry (blue bar, at around 4 weeks, 8 weeks, and 12 weeks of age on regular chow and between diet weeks 21–24 on custom diets), in vivo micro-computed tomography (purple triangle, 1–2 days before euthanasia). At euthanasia (red triangle), blood was collected, soft tissues were isolated, weighted, and snap-frozen, long bones were collected, length measured, and prepared for ex vivo micro-computed tomography. Mouse developmental stages:^68,69^. Created in BioRender. https://BioRender.com/zv11w9o

To generate OI mice lacking OCN, heterozygous *Col1a1*^*Jrt/+*^ mice (OI; FVB background) were bred with heterozygous *Bglap*^*+/-*^ mice (OCN; C57BL6J background), resulting in *Col1a1*^*Jrt/+*^/*Bglap*^*+/-*^ (OI/OCN-Het) and *Col1al*^*+/+*^/*Bglap*^*+/-*^ (OCN-Het) offspring on a mixed FVB/C57BL6J background. Next, OI/OCN-Het were crossed with each other to produce *Col1a1*^*Jrt/+*^/*Bglap*^*-/-*^ mice (OI/OCN-KO) (Fig. 1). In total, 294 female and 129 male mice were used to produce 10 consecutive generations (F1 to F10). Of these breeding pairs, 87% (*n* = 255) were successful, while 13% (*n* = 39) did not result in pregnancies. The 255 successful pairs resulted in total in 1 680 pups (F1 to F10), with an average litter size of 7 (ranging from 1 to 12). Among the 1 680 pups, 6.3% (*n* = 106) died within the first 1–3 days post-birth. Surviving mice from all generations exhibited normal phenotypic characteristics and demonstrated viability.

The phenotype of all mice born with a genetic mutation in *COL1A1* was similar to that of the original *Col1a1*^*Jrt/+*^ mouse colony, depicting reduced size, reduced body mass, and skeletal fragility. In the F1 generation, the genetic distribution was approximately 13% WT and OI, 33% OI/OCN-Het, and a 2% mortality rate (Table S1). In subsequent generations (F2 onward), the distribution shifted to approximately 8% WT, 16% OI, 26% OI/OCN-Het, 17% OI/OCN-KO, and 10% mortality. Observed deaths occurred across all genotypes and generations, with many linked to craniofacial abnormalities and brain hemorrhages, which impaired the nursing of the pups.

To confirm OCN reduction in OI mice, a subset of mice was analyzed for serum OCN levels (Table S2). As expected, and as previously shown,^22^ male and female OI mice with an intact OCN gene exhibited increased tOCN, GLA13-OCN, and GLU-OCN compared to sex-matched WT mice on a mixed background. OI/OCN-Het mice showed a significant reduction in serum OCN levels for all three isoforms (Table S2), comparable to serum levels in OCN-Het mice.

However, given that the mouse models are on different background strains, we explored if there is an indication of a potential strain impact by observing the fur color of the born offspring (Table S3). Mice with an exclusive FVB background display white fur, whereas those with a C57BL6 background exhibit black fur. In the F1 generation, crossbreeding consistently produced grey fur, implying a uniform FVB/C57BL6 expression. However, fur color variation emerged in later generations, suggesting potential variations in background strain expression. We performed correlation analyses of fur color per mouse per generation on all reported study’s outcomes, with no significant finding (*data not shown*). Thus, the study’s outcomes from all generations (F1 to F10) were combined for analysis and are presented here.

### OI breeding efficiency was not affected by osteocalcin deficiency

Previous reports indicated that OCN deficiency may affect fertility, primarily in males.^26,27^ Thus, we analyzed the breeding efficiency of male and female OI/OCN-KO mice during the generation of the mouse colony (F1-F10 generation). A total of 7 male OI/OCN-KO mice were crossed with 17 females of different genetic phenotypes. All male OI/OCN-KO mice successfully fathered pups, resulting in 75 offspring, with an average litter size of 4 pups (ranging from 1 to 8). Five pups ( ~6.7%) died during the first week after birth. A total of 27 OI/OCN-KO female mice were used for breeding, of which 81% (*n* = 22) were successful, while 19% (*n* = 5) were not. A total of 120 pups were born, and seven pups died (5.8%) during the first day after birth. The average litter size was 5, ranging from 2 to 9 pups. The breeding efficiencies of OI/OCN-KO mice were comparable to the breeding of male and female OI mice or OI/OCN-Het mice but lower than that of WT mice (Table S4). In summary, OCN deficiency did not affect male or female breeding efficiency in OI; however, diminished litter size was noted to be produced by male OI/OCN-KO mice, consistent with the previous report of reduced litter size by male OCN-KO mice.^26^

### Osteocalcin impact on development and metabolic organs is age- and sex-dependent in OI mice

**Fig. 2:**
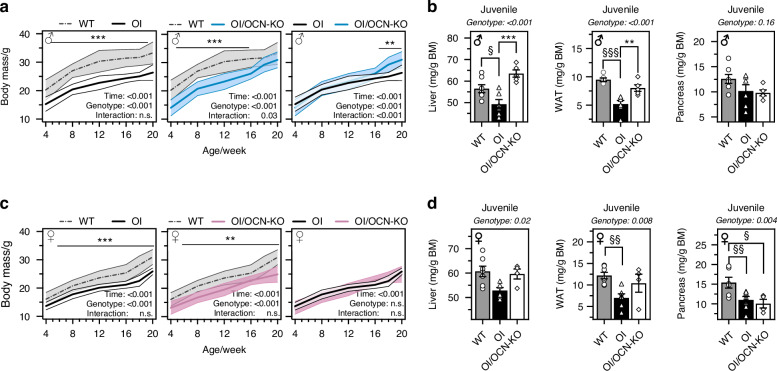
Body mass development in male and female (**a**, **c**) and organ masses (**b**, **d**) in WT and OI mice with regular and deficient OCN on regular chow. Data are mean ± SD (**a**, **c**) or mean ± SEM and individual measurements per genotype (**b**, **d**). Inguinal white adipose tissue masses are depicted. BM, body mass. Statistical analysis: **a**, **c** two-way ANOVA to assess overall effect of genotype, time, and interaction, followed by Bonferroni post-test to assess genotype effect per age: *P < 0.05, **P < 0.01, ***P < 0.001. **b**, **d** one-way ANOVA within each sex to assess for overall genotype effect, followed by Bonferroni post-test to assess OCN effect by comparison to OI only: **P < 0.01, ***P < 0.001; and assess OI and OCN effect by comparison to WT only: § *P* < 0.05, §§ *P* < 0.01, §§§ *P* < 0.001

Previous studies have reported increased body mass and fat mass in 3- to 9-month-old OCN-deficient mice.^15,28^ Based on this, we first compared WT, OCN-Het, and OCN-KO mice. However, we found no differences in body mass, tissue weights, or metabolic parameters among these groups (Figs. S1–3**)**. Therefore, we focused our analysis on WT and OI mice as we previously demonstrated lower fat masses in OI mice.^22^ Consequently, we assessed the overall development and organ masses of OI mice with altered OCN genetics on regular chow. Compared to sex-matched WT counterparts, the overall body mass development of male and female OI mice with regular, reduced, or absent OCN was significantly (s)lower (Fig. 2a, c; Table S5). However, when comparing OI, OI/OCN-Het, and OI/OCN-KO mice, the overall body mass development did not differ, except for male OI/OCN-KO mice, which exhibited significantly higher body mass with age, starting at around 16 weeks (Fig. 2a).

While overall development in OCN-deficient mice resembled OI mice, notable age- and sex-specific differences in organ masses emerged (Fig. 2b, d**;** Table S6). In juvenile males and females, a significant genotype effect was observed for liver and inguinal WAT masses, with OI mice exhibiting reduced organ masses compared to WT (Fig. 2b, d). These reductions were reversed in OI/OCN-KO mice (Fig. 2b, d). Additionally, juvenile female OI mice showed decreased pancreatic mass, a change that persisted despite OCN deficiency (Fig. 2d). With age, however, organ masses in both OI and OI/OCN-KO mice largely normalized (Table S6). These findings suggest that OCN deficiency may modulate early-life alterations in liver and inguinal WAT, but not pancreas, masses in OI mice, with most differences diminishing in adolescence.

### Osteocalcin deficiency does not improve OI long bone structural or material properties

Previous studies demonstrated greater bone strength and density in OCN-KO mice compared to their control littermates,^29^ thus, we assessed whether altered OCN levels further alter the bone phenotype in OI mice. At 4 weeks of age, no OCN-related effects were observed in OI/OCN-Het or OI/OCN-KO compared to OI mice (*data not shown*). As mice aged, a trend towards increased bone volume fraction (BV/TV) was observed, driven by a higher trabecular number (Tb.N.) in OI mice with reduced OCN levels only, but did not reach statistical significance (Table S7).

### On regular chow, osteocalcin modulates glucose homeostasis in OI in an age- and sex-dependent manner, while energy expenditure is OCN-independent

Previously, we demonstrated altered insulin and glucose homeostasis along with reduced adiposity and high energy expenditure in OI mice on regular chow.^22^ Additionally, OCN-KO mice have been shown to exhibit hyperglycemia, hypoinsulinemia, impaired insulin secretion and sensitivity, reduced glucose tolerance, and lower energy expenditure.^15^ Building on these findings, we examined glucose metabolism, glucose tolerance, and energy expenditure in OI/OCN mice on regular chow.

**Fig. 3:**
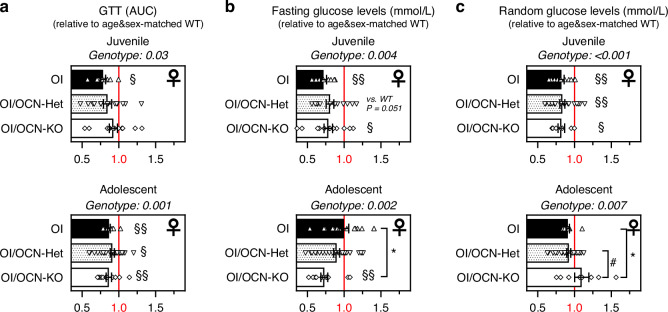
Glucose metabolism in female OI/OCN mice on regular chow at juvenile and adolescent age. Juvenile, 4 weeks of age. Adolescent, 8–12 weeks of age. **a** Area under the curve (AUC) for glucose tolerance tests (GTT), normalized to age- and sex-matched WT levels (1.0, red line). **b** Fasting glucose levels (mmol/L) at time point 0 of the GTT, normalized to age- and sex-matched WT levels (1.0, red line). **c** Random glucose levels (mmol/L), normalized to age- and sex-matched WT levels (1.0, red line). Absolute measurement values are listed in Tables S7, S8. Data represent fold-change from age- and sex-matched WT levels (set to 1.0, red line), and are depicted as means ± SEM, and individual measurements per genotype. Statistical analysis: One-way ANOVA to assess for overall genotype effect, followed by Bonferroni post-test to assess OCN effect by comparison to OI: *P < 0.05 or comparison of OCN-levels: #, *P* < 0.05; or assess OI and OCN effect by comparison to WT only: § *P* < 0.05, §§ *P* < 0.01, §§§ *P* < 0.001

Consistent with previous findings, OI mice displayed improved glucose metabolic parameters compared to WT (Fig. 3a–c, Fig. S4b, Tables S8,9). Specifically, juvenile female OI mice exhibited significantly enhanced glucose tolerance and lower random and fasting glucose levels relative to WT (Fig. 3a–c; Fig. S4b). In juvenile female OI mice lacking OCN, glucose tolerance returned to WT measures (Fig. 3a), but fasting and random glucose levels remained similar to OI mice (Fig. 3b, c). In adolescent age, glucose tolerance remained significantly improved in OI mice relative to WT independently of OCN levels (Fig. 3a). Of interest, in adolescent OI/OCN female mice, fasting glucose levels were significantly lower than both WT and OI mice (Fig. 3b), whereas random glucose levels were significantly higher than both WT and OI mice (Fig. 3c). In male OI mice, OCN deficiency primarily influenced random glucose levels (Fig. S5c).

Given our previous findings of sex-dependent differences in metabolic substrate utilization in young male and female OI mice,^22^ we conducted indirect calorimetry in OI and OI/OCN-KO mice. Notably, as previously observed, mice with the OI genotype exhibit significantly altered locomotion compared to their WT counterparts, likely due to spontaneous fractures and pain.^22,30^ To reduce variability caused by differences in activity levels, we first identified periods of low (LOMO) and high mobility (HIMO) for each genotype and used these time points for further analysis (Table S22**)**. Additionally, to isolate the effects of OCN, indirect calorimetry data were normalized to age -, sex -, and mobility-matched WT mice (Figs. S4c, S5d, Table S10). Juvenile and adolescent male and female OI and OI/OCN-KO mice demonstrated pronounced high VCO₂, VO₂ and energy expenditure, compared to WT, both at low and high mobility levels (LOMO and HIMO) (Figs. S4c, S5d). No significant effects of OI or OCN genotype on food intake were observed. However, an OCN-dependent effect on substrate utilization (as indicated by the respiratory exchange ratio, RER) was observed in juvenile male OI/OCN-KO mice, which diminished with age (Fig. S5d). Thus, under a regular chow diet, OCN modulates glucose homeostasis in OI mice, with distinct age- and sex-dependent effects, while increased energy expenditure in OI mice is OCN-independent.

### Male OI mice with osteocalcin deficiency improve insulin metabolism but lose protection against high-fat diet-induced obesity

**Fig. 4:**
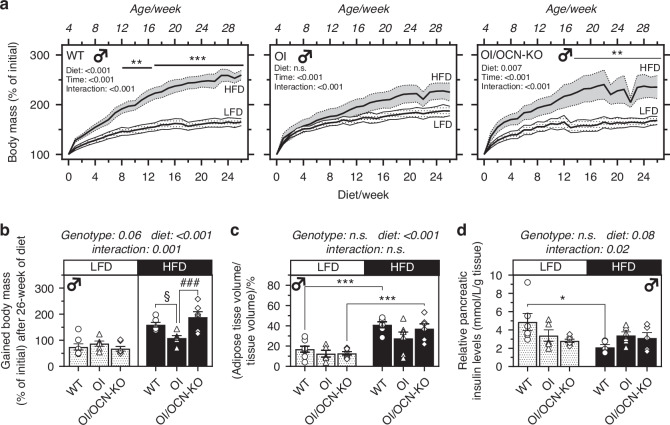
Obesity development in low-fat (LFD) or high-fat diet (HFD) fed male WT, OI and OCN-KO mice. **a** Body mass progression as %-change from the start of dietary intervention during the 26 weeks on LFD or HFD. **b** Gained body mass after 26 weeks of LFD or HFD. **c** Adiposity (adipose tissue volume over tissue volume) after 26 weeks of LFD or HFD. Data are mean ± SD (**a**) or mean ± SEM and individual measurements per genotype (**b**–**d**). Statistical analysis: **a** two-way ANOVA of repeated measures assessing the overall effect of diet, time, and their interaction, followed by Bonferroni post-test to assess diet effect: **P < 0.01, ***P < 0.001. **b**–**d** Two-way ANOVA assessing the overall effect of genotype, diet, and their interaction, followed by Bonferroni post-test (**b**) within each diet to assess genotype effect by comparison to diet-matched WT: § *P* < 0.05, and to diet-matched OI: ### *P* < 0.001. **c**, **d** to assess diet effects: *P < 0.05, **P < 0.01, ***P < 0.001, genotype effect by comparison to diet-matched WT: n.s., and to diet-matched OI: n.s

Previously, we observed sex-dependent metabolic differences in OI mice subjected to a long-term high-fat diet (HFD), with male OI mice being protected from HFD-induced obesity.^23^ To explore whether this protective effect is mediated by elevated OCN levels, we examined the metabolic phenotype of male OI and OI/OCN-KO mice after extended HFD or control low-fat diet (LFD) exposure. The obesity protection observed in male OI mice on HFD was lost in OCN-deficient male OI mice, as they developed obesity over time on HFD, similar to WT mice (Fig. 4a) and gained body mass of about +104% on HFD compared to LFD (Fig. 4b, Tables S11, S13). Conversely, adiposity, a measure of visceral and subcutaneous fat pads, was similar to WT in OI/OCN mice on HFD (Fig. 4c, Table S14). Interestingly, in our prior study using a pure FVB background, female OI mice with unaltered OCN genetics developed HFD-induced obesity and elevated fasting glucose levels over time.^23^ HFD-induced obesity was not as strongly replicated in female OI mice on a mixed background (Fig. S6a), suggesting that the presence of the C57BL6 background may have positively influenced the metabolic phenotype of female OI mice. Nonetheless, both female OI and OI/OCN-KO mice gained body mass and exhibited similar adiposity levels after 26 weeks of HFD (Fig. S6b, Tables S12, S13).

**Fig. 5:**
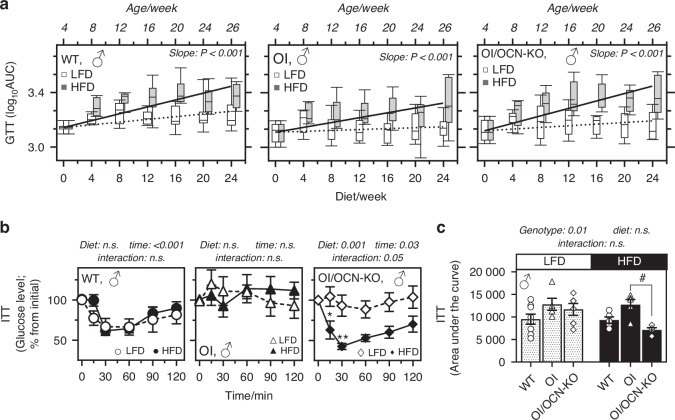
Glucose and insulin homeostasis in low-fat (LFD) or high-fat diet (HFD) fed male WT, OI and OCN-KO mice. **a** Glucose tolerance test (GTT) was performed before ( =0) and at 4, 8, 12, 16, 20, and 24 weeks on LFD or HFD; the area under the curve (AUC) was quantified and log_10_ transformed. Data are represented as box plots indicating median and 10th/90th percentile per time point. **b** Insulin tolerance tests (ITT) after 26 weeks of diets, shown as changes in glucose levels (% from initial glucose levels) after insulin injection at time point 0. Data are presented as mean ± SEM per time point. **c** Average AUC of ITT presented in (**b**). Data are mean ± SEM and individual measurements per genotype. Statistical analysis: **a** Non-linear regression analysis with evaluation of statistical differences between curve slopes. **b** two-way repeated measures ANOVA assessing the overall effect of diet, time, and their interaction, followed by Bonferroni post-test to assess diet effect per timepoint: *P < 0.05, **P < 0.01. **c** two-way ANOVA assessing the overall effect of genotype (**a**), diet (**b**), and their interaction (**c**), followed by Bonferroni post-test to assess the effect of diet (n.s.) and genotype compared to diet-matched OI: # *P* < 0.05 or WT: n.s

On HFD, mice of all genotypes exhibited glucose intolerance and high fasting glucose (Fig. 5a, Figs. S6d, S7, Table S15). Correlation analysis indicated that at the end of the dietary intervention, GTT was associated with adiposity in male WT (*P* = 0.08), OI (*P* = 0.001), and OI/OCN-KO mice (*P* < 0.001) (Fig. S8a). Notably, OI/OCN-KO mice seemed to exhibit higher GTT on HFD than OI mice (Fig. S7b). Insulin tolerance testing (ITT) revealed a significant effect of OCN on insulin sensitivity in OI mice, with male OI/OCN-KO mice showing ITT responses similar to WT under HFD conditions (Fig. 5b, c, Tables S16, S17). Consistent with our previous findings,^23^ female OI and OI/OCN-KO mice exhibited elevated fasting glucose levels and progressive glucose intolerance on HFD (Figs. S6d, S7c). Correlation analysis revealed that GTT outcomes were linked to adiposity in female WT (*P* = 0.002), OI (*P* < 0.001), and OI/OCN-KO mice (*P* < 0.001). Additionally, GTT correlated with body mass in female WT and OCN-deficient mice but not in OI mice (Fig. S8h).

Absolute pancreatic insulin levels demonstrated a significant overall genotype difference, with male OI and OI/OCN-KO mice displaying lower levels compared to WT (Table S18). When normalized to pancreatic mass (Table S5), insulin levels revealed a significant diet-driven interaction with HFD-fed WT mice exhibiting reduced insulin levels compared to LFD-fed WT mice, while insulin levels in OI and OI/OCN-KO mice remained unchanged (Fig. 4d, Table S18). In female mice, absolute pancreatic insulin levels were not significantly different among genotypes (Table S18); however, normalized insulin levels showed a similar pattern to males, with WT mice having lower insulin levels on HFD, while OI mice showed an increase, and OI/OCN-KO mice remained unchanged. These results indicate that pancreatic insulin levels in OI mice are independent of OCN. Moreover, ITT responses did not correlate with adiposity, body mass gain, or normalized pancreatic insulin levels (Fig. S8b, d, e for male mice, S8g, i, j for female mice).

These findings highlight a sex-dependent role of OCN in adipose metabolism and insulin responses in OI mice. While OCN deficiency disrupted obesity protection and altered insulin responses in male OI mice, its effects on metabolic parameters in female OI mice were less pronounced and influenced by genetic background. The pancreatic insulin production was altered in OI mice of both sexes in an OCN-independent manner.

### Osteocalcin drives energy expenditure in high-fat diet-fed OI mice

Previously, we reported increased energy expenditure in male OI mice subjected to long-term HFD,^23^ which correlated with OCN serum levels, suggesting a possible causality. Hence, we examined whole-body energy expenditure in OI and OI/OCN-KO mice during long-term dietary intervention. Of note, as observed before, mice with the OI genotype exhibit significantly altered locomotion compared to their WT counterparts, likely due to spontaneous fractures and pain.^22,30^ Thus, we first identified periods of low (LOMO) and high mobility (HIMO) for each genotype to reduce variability from activity levels (Table S23), and indirect calorimetry data were normalized to age -, sex -, and mobility-matched WT mice.

**Fig. 6:**
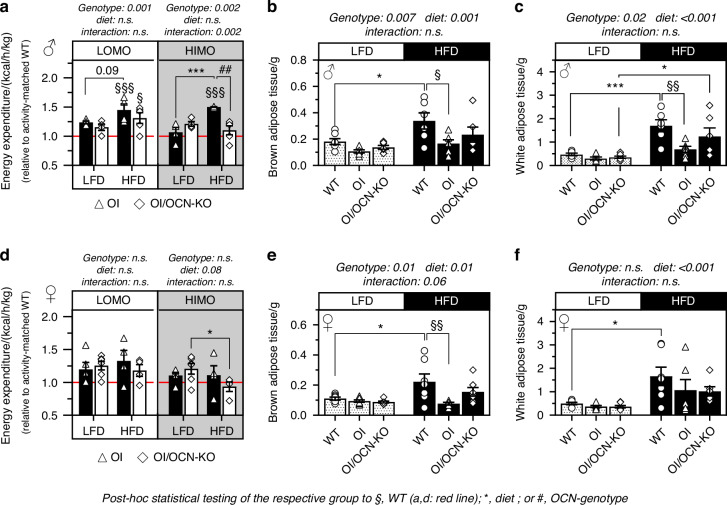
Relative energy expenditure of male (**a**) and female (**b**) OI and OI/OCN-KO mice on LFD or HFD, and absolute organ masses (**b**, **e**, **c**, **f**) of WT, OI, and OI/OCN-KO mice after long-term LFD or HFD. Data indicate single measurements and mean ± SEM. LFD, low-fat diet. HFD, high-fat diet. LOMO, low mobility levels. HIMO, high mobility levels. All measurements were normalized to age-, sex- and mobility-matched WT mice and are presented as a fold-change from WT (set to 1.0, red line). Statistical analysis: (**a**, **d**) two-way ANOVA within each sex and mobility to assess the overall effects of genotype, diet, and their interaction; followed by Bonferroni post-hoc testing for diet effects: *P < 0.05, ***P < 0.001; and genotype effect by comparison to diet- and mobility-matched WT: § *P* < 0.05, §§§ *P* < 0.001, or diet- and mobility-matched OI: ##, *P* < 0.01. (**b**, **c**, **e**, **f**) two-way ANOVA assessing the overall effect of genotype, diet, and their interaction, followed by Bonferroni post-test to assess diet effects: * *P* < 0.05, ** *P* < 0.01, *** *P* < 0.001, genotype effect by comparison to diet-matched WT: § *P* < 0.05, §§ *P* < 0.01, and to diet-matched OI: n.s

We observed sex- and mobility-dependent effects of OCN on energy expenditure under HFD conditions. Male OI mice on HFD demonstrated higher VO₂, VCO₂ and energy expenditure compared to WT during periods of high mobility, and OCN deficiency significantly reduced VCO₂ and VO₂ and energy expenditure (Fig. 6a, Fig. S9a, b). In contrast, in female mice VO₂, VCO₂ and energy expenditure were similar in WT, OI and OI/OCN mice (Fig. 6d, Fig. S9f, g). Respiratory exchange ratio (RER) and relative food intake were not significantly affected by diet or genotype in both sexes (Fig. S9c, d, h, i).

The sex-dependent metabolic patterns aligned with differences in body surface temperature regulation (Tables S20, S21). In males, the body surface temperature of WT and OI/OCN mice was similar to that of LFD and HFD; however, male OI mice with normal OCN levels maintained higher body surface temperatures on HFD than on LFD (Fig. S9e). In female mice, body surface temperature was diet-independent in WT but higher on HFD in both OI and OI/OCN mice (Fig. S9j).

The primary driver of energy expenditure is thermogenesis, which is largely regulated by BAT activity.^31^ Interscapular BAT mass increased following HFD in WT and OI/OCN mice of both sexes; however, the diet-induced effect was smaller in male OI mice and non-existent in female OI mice (Fig. 6b, e, Table S22). Since BAT activity is influenced by signals from other tissues, such as white adipose tissue (WAT), the liver, brain, and muscle,^32^ we also examined these metabolic tissues. Absolute inguinal WAT mass significantly increased following HFD in WT, OI, and OI/OCN mice of both sexes in a genotype-specific manner (smaller increases in OI mice) (Fig. 6c, f, Table S22). Liver or gastrocnemius muscle masses were not affected by diet but were affected by genotype (lower in OI and OI/OCN) (Table S22). In summary, OCN-mediated energy regulation appears to contribute to obesity resistance in male OI mice.

## Discussion

**Fig. 7:**
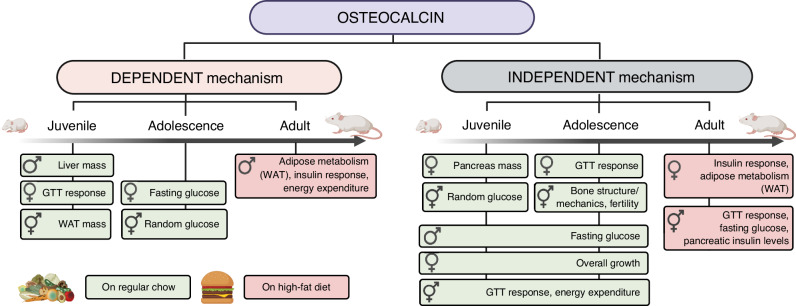
Summary of osteocalcin-dependent and -independent mechanisms in a mouse model mimicking severe Osteogenesis imperfecta. GTT, glucose tolerance testing; WAT, inguinal white adipose tissue. Created in BioRender. https://BioRender.com/zv11w9o

Previously, we demonstrated that mice with severe dominant OI exhibit an age- and sex-dependent metabolic phenotype,^22^ which correlated with high serum levels of bone-derived endocrine hormone OCN. Challenging these mice with a long-term HFD uncovered the protection of male OI mice from diet-induced obesity, which was associated with altered OCN levels.^23^ Now, using genetic inactivation of OCN in OI *Col1a1*^*Jrt/+*^ mice, we identified both OCN-dependent and OCN-independent mechanisms contributing to the metabolic phenotype in OI (Fig. 7).

Specifically, in young OI mice under regular chow conditions, OCN-dependent metabolic aspects included adipose and liver development, as well as ‘fine-tuning’ glucose homeostasis in an age- and sex-dependent manner. Under HFD, our data demonstrate a particularly important role of OCN in male OI mice, where it mediated diet-induced weight gain by adapting adipose metabolism, insulin resistance, and energy expenditure, thereby potentially protecting male OI mice against diet-induced obesity. In contrast, OCN-independent traits in OI included energy expenditure and pancreas development in juvenile mice under regular chow conditions, as well as glucose homeostasis and pancreatic insulin production in HFD-challenged adult OI mice. These findings suggest that the metabolic phenotype in OI mice is complex, driven both by OCN, likely elevated due to high bone remodeling, and by OCN-independent mechanisms that warrant further investigation.

OCN-dependent metabolic regulation was apparent in metabolic organ development in juvenile OI mice; however, it was particularly evident in the context of HFD, especially in male OI mice. These animals showed altered glucose and insulin metabolism, fat accumulation, and energy expenditure, with OCN deficiency reversing many of these phenotypes. While HFD-fed male OI mice developed glucose intolerance and insulin resistance over time, the absence of OCN led to improved insulin sensitivity despite persistent glucose intolerance. This finding is paradoxical as HFD generally leads to the simultaneous development of obesity, glucose intolerance, and insulin resistance.^33–35^ OCN-deficient OI mice fed a HFD exhibited improved insulin sensitivity, increased pancreas mass, and a trend toward elevated pancreatic insulin content. These results challenge a bit the hypothesis of OCN as a regulator of insulin production and sensitivity. Previously, OCN-deficient mice on C57BL6 background demonstrated impaired insulin secretion and reduced insulin sensitivity under normal chow conditions^15^; and that OCN–Gprc6A signaling is critical for late pancreatic morphogenesis and β-cell development.^36^ In contrast, daily OCN injections in HFD-fed WT mice reduce circulating insulin levels while improving insulin sensitivity, suggesting a role in enhancing insulin efficiency rather than absolute production.^37^ Furthermore, it has been shown that OCN exerts concentration-dependent effects, with low ucOCN levels stimulating insulin gene expression and β-cell proliferation, while higher ucOCN levels suppress insulin transcription and increase adiponectin expression in adipocytes.^16^ Given these findings, worsened metabolic outcomes would be expected in OCN-deficient OI mice, particularly under HFD. However, our data indicate that in the context of OI on a mixed background, insulin sensitivity can improve despite the absence of OCN, possibly through compensatory mechanisms that bypass OCN-dependent pathways. These findings highlight the complexity of OCN’s role in metabolic regulation, which appears to be highly nuanced and context-specific. For instance, glucose tolerance seems OCN-independent in adolescent females, whereas fasting and random glucose levels at the same stage appear OCN-dependent. This may indicate that OCN contributes more strongly to basal glucose homeostasis (reflected by fasting and random glucose levels) than to the acute response to a bolus glucose challenge. Moreover, the role of OCN may be further influenced by life stage, hormonal changes, and dietary conditions, suggesting that additional, yet unidentified factors may sustain glucose/insulin homeostasis in its absence. Further research is needed to fully uncover the potential mechanism underlying these observations.

In addition to its role in insulin regulation, OCN mediated changes in fat metabolism and energy expenditure under HFD, especially in males. Under HFD, male OI/OCN-KO mice exhibited reduced energy expenditure during periods of high mobility, suggesting impaired BAT and/or muscle thermogenesis. This reduction contributed to increased fat accumulation, supporting a role for OCN in promoting energy dissipation under metabolic stress. In line with this, daily OCN injections in HFD-fed WT C57Bl/6J mice were shown to increase mitochondrial number, potentially enhancing energy expenditure.^37^ Additionally, ucOCN has been reported to promote glucose uptake in oxidative muscle fibers.^38^ Conversely, thermogenesis under regular chow appeared OCN-independent and may be driven by sympathetic activation, thyroid hormones, or environmental factors such as cold exposure.^39–41^ For instance, mice were housed at room temperature (21-22 °C) and must increase energy expenditure to maintain body temperature, potentially enhancing BAT activity.^40,41^ Together, these findings underscore the multifaceted role of OCN in coordinating metabolic adaptations to dietary stress, particularly in males, and reveal previously unrecognized compensatory mechanisms that may buffer against its loss in the context of OI.

OCN-independent metabolic traits were evident both at baseline and under HFD. Juvenile OI mice exhibited enhanced energy expenditure and altered pancreas development regardless of OCN status, suggesting intrinsic metabolic changes in OI. In HFD-fed adult mice, glucose dysregulation and elevated pancreatic insulin content persisted in the absence of OCN, implicating additional disease-related factors. Dyslipidemia, previously reported in OI mice,^23^ characterized by elevated cholesterol and altered lipoprotein profiles, may contribute to insulin resistance.^40,42^ While OCN has been linked to lipid regulation via modulation of SREBP1 and the mevalonate pathway,^43–45^ the persistence of these traits in OCN-deficient mice suggests involvement of alternative regulators. We have also found that in OI mice, overall growth, bone metabolism and fertility are OCN-independent, even though these physiological parameters were previously demonstrated to be regulated by OCN.^46^ These observations suggest that the collagen-I mutation underlying OI, by altering the function of osteoblasts as both bone-forming and hormone-secreting cells, exerts wide-ranging effects on bone metabolism, growth, and fertility that surpass and potentially overshadow the regulatory contributions of OCN.

Further, sexual dimorphism in OI metabolism was evident across diets and genotypes. Female OI mice demonstrated improved glucose tolerance in adolescence and stable insulin sensitivity under HFD. These milder metabolic alterations may relate to lower circulating OCN levels in females,^23^ along with hormonal and chromosomal influences. Of interest, and consistent with our previous findings,^22^ we observe sex-dependent differences in OCN effects in juvenile mice, in which the direct effect of sex hormones is not yet established. Testosterone can activate the OCN receptor GPRC6a, enhancing BAT thermogenesis and lipid uptake.^47,48^ Furthermore, androgen signaling through hepatic and pancreatic androgen receptors may influence bioenergetics.^49^ Differences in sex chromosome dosage also affect lipid metabolism, with HDL and LDL levels modulated by X and Y chromosomes, respectively.^50^ Generally, X chromosome gene expression is balanced between XX and XY cells through one X chromosome inactivation in females, but some genes escape this inactivation, leading to higher expression in XX compared to XY cells.^51^ These factors may underlie the distinct metabolic phenotypes observed between male and female OI mice.

While the metabolic phenotype observed in *Col1a1*^*Jrt/+*^ mice may not directly translate to human OI, a key observation of this study is the age-dependency of metabolic alterations in OI. The effects of OCN were most pronounced during early developmental stages and less evident in adulthood under baseline conditions, paralleling physiological changes in bone turnover.^6^ In OI, this process is amplified due to the underlying collagen-I mutation leading to increased osteoblast activity, resulting in heightened bone turnover, especially in young OI patients and OI mice.^6,22^ In keeping, circulating OCN levels were reported to be high in early life and decrease with age in mice and patients with OI,^22,52^ with corresponding evidence of hypermetabolism in young OI mice^22^ and pediatric OI patients.^11^ Anthropometric trends further support an age-related shift in OI metabolism. Young children with OI commonly present with low body weight for height, especially before puberty,^53–55^ and weight gain has also been reported as a side effect of pamidronate therapy, particularly in male pediatric patients.^56^ In contrast, older OI patients often become overweight or obese.^14,54,57^ We demonstrate that under metabolic stress, such as HFD, OCN signaling re-emerged as a significant modulator of metabolic phenotype in OI mice. Although systematic studies are lacking in OI patients, case reports suggest that individuals with OI may be predisposed to diabetes,^58,59^ raising the possibility that OI patients may develop features of metabolic syndrome, such as insulin resistance and obesity, later in life. Together, these findings reinforce the need to consider the developmental stage in the metabolic evaluation of OI patients.

However, the precise mechanisms by which OCN exerts observed effects on metabolism in OI still require further investigation using additional OI mouse models. For instance, studies using other models such as oim/oim mice under varied dietary conditions will be essential to determine whether OCN directly regulates metabolic processes or whether the observed effects arise from secondary adaptations in body composition, energy expenditure, or other OI-associated traits. It is also important to note that studies have reported an inability to reproduce the endocrine functions of OCN,^18–20^ suggesting that its metabolic effects may depend on factors such as mouse strain, age, and sex. Previous research has primarily involved 129/Sv, C57BL6N, or C57BL6J mice, often focusing on single-sex and different age groups ranging from 10 weeks to 12 months. In contrast, our study included both sexes, young and adolescent mice, applied regular chow and custom diets, and involved a mixed FVB/C57BL6 genetic background.

In conclusion, this study validates the role of OCN as a key modulator of metabolism in OI and identifies both OCN-dependent and -independent contributors to the phenotype. We demonstrate that metabolic abnormalities are most prominent during juvenile stages but can persist or re-emerge under stress in adulthood. Our findings reveal complex metabolic dysregulation in OI and underscore the need to consider age- and sex-specific OCN pathways in developing personalized diagnostics and broader management strategies that address the multi-system nature of the disease.

## Materials and methods

### Animals

All experiments were approved by the Animal Care Committee of McGill University, complied with ARRIVE guidelines, and conformed to the ethical guidelines of the Canadian Council on Animal Care. The *Col1a1*^*Jrt/+*^ mice on the FVB background were a gift from Jane Aubin (University of Toronto, Toronto, Canada). *Col1a1*^*Jrt/+*^ mice carry a splice site mutation in exon 9 of the *COL1A1* gene, leading to an 18-amino acid deletion in the collagen type I α1 chain.^21^ *Col1a1*^*Jrt/+*^ mice are smaller in size, have lower bone volume/tissue volume (BV/TV), develop spontaneous fractures, and have reduced muscle mass,^21,60^ mimicking moderate-to-severe forms of OI. *Col1a1*^*Jrt/+*^ genotype is always heterozygous, as homozygosity is lethal.^21^ Osteocalcin (*Bglap*^*–/–*^) knock-out mice on C57BL6J background were generated by replacing *Ocn1* (*Bglap1*) and *Ocn2* (*Bglap2*) genes in the mouse *Ocn* cluster with a neomycin resistance cassette through homologous recombination.^29^ Breeding colonies were maintained at the Shriners Hospitals for Children-Canada Animal Care Facility. If not indicated otherwise, all animals were on a 12-h alternating light and dark cycle and had unrestricted access to water and regular chow.

### OCN-deficient *Col1a1*^*Jrt/+*^ mice

The *Col1a1* gene is located on murine chromosome 11 while *OCN* gene clusters are located on chromosome 3. To generate OCN-deficient *Col1a1*^*Jrt/+*^ mice (OI/OCN), first *Col1a1*^*Jrt/+*^ mice (OI; FVB background) were bred with *Bglap*^*+/-*^ mice (OCN; C57BL/6 J background) to generate *Col1a1*^*+/+*^/*Bglap*^*+/+*^ (WT/WT), *Col1a1*^*Jrt/+*^/*Bglap*^*+/+*^ (OI/OCN-WT), *Col1a1*^*Jrt/+*^/*Bglap*^*+/-*^ (OI/OCN-Het) and *Col1a1*^*+/+*^/*Bglap*^*+/-*^ (WT/OCN-Het) mice of mixed FVB/C57BL/6J background (Fig. 1a). To generate mice with a *Col1a1*^*Jrt/+*^/*Bglap*^*-/-*^ genotype (OI/OCN-KO), OI/OCN-Het and OI/OCN-Het mice were crossed. Subsequently, the colony was maintained by crossing of OI/OCN-KO and OI/OCN-Het mice with OI/OCN-KO or OI/OCN-Het mice from the same generation to keep the contributions of the FVB and C57BL/6J backgrounds as constant as possible. The phenotyping experiments were performed in mice generated over 10 generations (F1 to F10) on mixed backgrounds. The following genotypes on mixed FVB/C57BL6J background were generated: *Col1a1*^*+/+*^/*Bglap*^*+/+*^ (WT/WT), *Col1a1*^*+/+*^/*Bglap*^*+/-*^ (WT/OCN-Het), *Col1a1*^*+/+*^/*Bglap*^*-/-*^ (WT/OCN-KO), *Col1a1*^*Jrt/+*^/*Bglap*^*+/+*^ (OI/WT), *Col1a1*^*Jrt/+*^/*Bglap*^*+/-*^ (OI/OCN-Het), *Col1a1*^*Jrt/+*^/*Bglap*^*-/-*^ (OI/OCN-KO). Figure 1a illustrates the breeding scheme. For the experiments described here, only mice with the genotypes *Col1a1*^*+/+*^*/Bglap*^*+/+*^, *Col1a1*^*Jrt/+*^/*Bglap*^*+/+*^, *Col1a1*^*Jrt/+*^/*Bglap*^*+/-*^, *Col1a1*^*Jrt/+*^/*Bglap*^*-/-*^, of the F1 to F10 generation, were used, referred to as WT, OI, OI/OCN-Het, and OI/OCN-KO mice, respectively. Of note, crossing OI/OCN-Het mice with OI/OCN-Het mice can also produce mice with *Col1a1*^*-/-*^genotype. However, a homozygous mutation in *Col1a1* is lethal; thus, these mice are not produced.

### GLU, GLA13, and total mouse osteocalcin ELISA

Total mouse osteocalcin (tOCN) and Gla13-OCN were measured in non-fasted serum using a specific ELISA assay as described previously.^22,61^ Briefly, ELISA plates (R&D system) were coated at room temperature with purified anti-Gla13-OCN or anti-MID-OCN antibodies diluted in 1X coating buffer (ImmunoChemistry Technologies) overnight. Following 2 washes with wash buffer (1X PBS, 0.1% Tween), blocking buffer (3% BSA, 1X PBS, 0.1% Tween, 0.05% sodium azide) was added for 4 h at room temperature. Blocking buffer was then replaced with general assay diluent (3% BSA, 1X PBS, 0.1% Tween, 0.05% sodium azide). For OCN measurements, Gla-OCN standard (0–200 ng/mL) or diluted serum samples were added to the assay diluent, respectively, into the plate. The plate was then sealed and incubated overnight at 4 °C. Following 5 washes, HRP-conjugated anti-CT-OCN was added and incubated for 1 h at room temperature. After several washes, TMB substrate (Pierce) was added and incubated for 15 min at room temperature. Reaction was stopped by adding stop solution (1 mol/L HCl), and absorbance was measured at 450 nm. Concentrations of Gla13-OCN and tOCN in the serum samples were calculated from a second-order polynomial obtained from the standard curve included in each assay. GLU-OCN was calculated by subtracting Gla13-OCN from tOCN.

### Dietary intervention

Male and female mice were randomly assigned to either (A) regular chow feeding (Teklad Chow Diet, #2918) to characterize the mouse model and assess general metabolic behavior; *OR* (B) a long-term custom diet intervention (custom-diet length: 26 weeks) feeding a high-fat/low-sugar diet (HFD; Teklad Custom Diet, #TD.06414) or control low-fat/low-sugar diet (LFD; Teklad Custom Diet, #TD.180127 - custom diet variant of #TD.08806), starting at an age of 4 weeks (Fig. 1b, c). Regular chow and custom diets were purchased from Envigo (Huntingdon, UK), and Table S24 lists their composition. Regular chow was supplemented with minerals, amino acids, vitamins (vitamin A, D_3_, E, K_3_, B_1_, B_2_, B_6_, B_12,_ Niacin, Pantothetic Acid, Biotin, Folate), and fatty acids (C16:0 Palmitic, C18:0 Stearic, C18:1ω9 Oleic, C18:2ω6 Lineoleic, C18:3ω3 Linolenic) and was stored at room temperature before usage. Custom diets were supplemented with mineral mix and vitamin mix (Niacin, Thiamin, Riboflavin, Folic acid, Biotin, vitamin B_12_, E, A, D_3_, K_1_) and stored at 4 °C before usage. Food was replaced once per week.

During the 26-week custom diet intervention, body mass was recorded weekly and gained body mass was calculated by subtracting body mass at diet start from body mass at diet week 1 to 26 for each individual (Fig. 1c). Body surface temperature at the anterior side under the diaphragm (PARAMED Infrared Thermometer, Model YK-IRT1, Prolinx GmbH, Duesseldorf, Germany) was documented weekly between 15-16 o’clock, glucose homeostasis was evaluated by performing glucose tolerance test (GTT) every 4-weeks, indirect calorimetry was evaluated between diet week 21 - 24 (age: 25 - 28 weeks), insulin tolerance test (ITT) at 26-weeks of diet, and adiposity was assessed by in vivo micro-computed tomography measurements 2 days before euthanasia (Fig. 1c).

Male and female mice fed regular chow were euthanized either at juvenile (4 weeks of age) or adolescent age (8 or 12 weeks of age). Male and female mice fed a long-term custom diet were euthanized at the end of the intervention at 30 weeks of age (adult, mature mice). All study-assigned mice were anesthetized using isoflurane inhalation and terminated by an intracardiac puncture to collect blood, followed by cervical dislocation. Blood serum was separated by centrifugation and stored at −80 °C until analysis. For mice fed regular chow, the following organs were isolated, weighed, snap-frozen in liquid nitrogen, and stored at −80 °C for later analysis: liver, pancreas, interscapular brown adipose tissue, and inguinal white adipose tissue. For mice undergoing a diet study, the same organs, along with the right gastrocnemius, were isolated, weighed, snap-frozen, and stored under the same conditions.

Dietary interventions and outcome measures were conducted as described above for male and female WT, OI, OI/OCN-KO, and OCN-KO mice. This manuscript focuses on the results from male and female WT, OI, and OI/OCN-KO mice. Outcomes from OCN-KO mice under long-term dietary intervention are available in refs. ^62,63^

### Glucose and insulin tolerance test

The glucose tolerance test (GTT) was performed at various ages (Fig. 1b, c). Before GTT, food was withdrawn for 5 h for mice younger than 6 weeks of age and 16 h for mice older than 6 weeks. Mice were injected with 200 μL of a sterile glucose solution (2 g/kg body mass) intraperitoneally. The insulin tolerance test (ITT) was performed in custom-diet-fed mice after 26 weeks of feeding. Before ITT, mice were fasted for 5 h and then injected intraperitoneally with 200 μL of sterile insulin-PBS solution (0.6 U/kg body mass insulin, Humulin® R, 100 U/mL, Lilly Pharmaceuticals). For GTT and ITT, blood glucose was measured using single-use glucose strips with the ONETOUCH VerioFlex glucometer (LifeScan Europe, Zug, Switzerland) in samples from the tail tip immediately before (time 0) and 15, 30, 60, 90, and 120 min after injection. ITT data are presented as a percentage of the initial blood glucose concentration. For GTT and ITT, the area under the curve (AUC) was calculated using the trapezoid rule.^64^ Furthermore, the AUC data for the GTT were log-transformed to normalize the data, improve homoscedasticity (equal variance of residuals), and stabilize variance.^65^

### Assessment of indirect calorimetry

Indirect calorimetry was assessed at 4, 8, and 12 weeks of age using the Oxymax/CLAMS Enclosure ITS2SD system (Columbus Instruments, Columbus, US). Oxymax/CLAMS measured O_2_ consumption, CO_2_ production, locomotion, and food intake from 12 parallel chambers each. Cages were enriched with a piece of paper (4 cm×4 cm) for nesting. Food and water were always provided ad libitum. Metabolic parameters were measured for 30 seconds after 90 seconds of cage settlement. Reference was measured every second chamber for 30 seconds after 90 seconds of reference settlement. Feeding measurements were performed synchronously with a metabolic assessment with a minimum idle time of 10 seconds and a minimum weight of 0.03 g. Unstable feeding events, negative measurements (e.g., mouse slept on the feeder), and measurements above 2.0 g (e.g., mouse removed food from the feeder without eating) were excluded from the analysis. Locomotion was assessed via infrared beams (one beam break is one count) with an activity sampling rate of 1 minute and a position sampling rate of 1 Hz. Mice were housed in the chambers for 4 consecutive days, where days 1 to 3 were used as the acclimatization phase for the mice to the chambers and day 4 for final analysis. Body mass was measured for every mouse on day 1 before being placed into the chambers and on day 5 after the experiment. Mice that lost more than 2 g of body mass were excluded from the final analysis due to these mice’s atypical feeding and drinking behavior. For analysis, metabolic, feeding, and locomotion measurements behavior were averaged for 4 day-time periods (06:00 am–08:59 am, period 1; 9:00 am - 11:59 am, period 2; 12 pm–2:59 pm, period 3; 03:00 pm–05:59 pm, period 4) and for 4 night-time periods (06:00 pm - 08:59 pm, period 5; 09:00 pm–11:59 pm, period 6; 12:00 am - 02:59 am, period 7; 03:00–05:59 am, period 8) of day 4. As observed before, mice with OI genotype exhibit significantly altered locomotion compared to their WT counterparts, likely due to spontaneous fractures and pain.^22,30^ In these studies, we identified distinct movement clusters in OI mice that do not align with the typical day/night activity cycles observed in WT mice. To minimize variations within measurements caused by differences in locomotion activity, we identified periods with activity levels of low mobility (LOMO) and high mobility (HIMO) for each genotype and dietary intervention. These periods were then used for further analysis of the metabolic phenotype. Table S23 depicts overall locomotion activity and indicates the analyzed periods. For each locomotion level, production of carbon dioxide (VCO_2_, mL/kg/h), consumption of oxygen (VO_2_, mL/kg/h), respiratory exchange ratio (VCO_2_/VO_2_), heat production (kcal/h/kg), and food consumption (g) were analyzed.

### Quantification of adiposity using in vivo micro-computed tomography

One to two days before euthanasia, mice were scanned using in vivo micro-computed tomography (Skyscan1276, Bruker, MA, US) under isofluorane/O_2_ anesthesia. Mouse was placed supine on a low-density polystyrene foam bed and scanned using the following parameters: voxel size of 42.6 μm, a 0.80-degree increment angle, 2 frames averaged, a 55 kV, and 200-mA X-ray source with a 0.5-mm Al filter (scanning time: 2-3 minutes). After reconstruction, adipose tissue volume was determined within the region of interest, ranging from the proximal end of vertebrae L1 to the distal end of vertebrae L5. Adipose tissue, referring to visceral and subcutaneous fat pads, was segmented according to Bruker Method Note MN027 “Quantifying adipose tissue (fat) in a mouse or rat by in-vivo microCT” and quantified using the system’s analysis software (Skyscan CT Analyser, Version 1.16.1.0).

### Ex vivo micro-computed tomography

Micro-computed tomography of the right femora were performed using Skyscan 1272 (Bruker, MA, US). Femoral scan parameters included a voxel size of 5 μm, a 0.40-degree increment angle, 3 frames averaged, a 66 kV and 142-mA X-ray source with a 0.5-mm Al filter to reduce beam-hardening artefacts. Trabecular bone was analyzed in a region starting at 0.5 mm proximal to the distal femoral growth plate (to avoid primary spongiosa) and scanning a section spanning 10% of the femoral length in a proximal direction. Trabecular bone was automatically segmented from the inner cortical surface and quantified using the system’s analysis software (Skyscan CT Analyser, Version 1.16.1.0). To analyze cortical bone, scanning was performed at the middle of the femur spanning a section of 5% of the femoral length. The software derives the outer bone diameter and the diameter of the bone marrow cavity from cross-sectional areas using a circular bone cross-section model. Cortical thickness was calculated as the difference between these two diameters divided by 2. Nomenclature and abbreviations follow the recommendations of the American Society for Bone and Mineral Research.^66^

### Three-point bending test

Following ex vivo micro-computed tomography scanning, the right femora were loaded to failure in three-point bending using a Materials Testing System Model 5943 (INSTRON, Norwood, MA, USA). The specimens were thawed one day before the test, and all muscle tissues were cleaned off. The bone was soaked overnight in 1X PBS at room temperature until mechanical testing. The distance between the lower supports was 7 mm with a vertical displacement rate of 50 μm/s. The anterior mid-diaphysis was loaded under tension. Test results were recorded using the system’s analysis software Bluehill (Illinois Tool Works Inc., Glenview, IL, USA; Version 3.65).

### Pancreatic insulin levels

Insulin levels were measured in pancreatic homogenates using an acidic-ethanol extraction method (Mercodia AB, Uppsala, Sweden, Technical Note #34-0137). A weighted fresh isolated pancreas was collected in acidified ethanol buffer (0.18 mol/L HCl, 70% (vol/vol) ethanol) and incubated overnight at -20 °C. Next, pancreas homogenate was generated using an Omni tissue homogenizer and incubated overnight at -20 °C. Afterwards, the homogenate was centrifuged at 3 200 x*g* for 15 minutes at 4 °C. The supernatant was then neutralized with 1 mol/L Tris pH 7.5 in a 1:1 ratio, and samples were stored at −80 °C until analysis. Insulin levels were determined in neutralized supernatants (diluted: 1:5 000 – 1:10 000) using Mouse Insulin ELISA (Mercodia AB, Uppsala, Sweden, # 10-1247-01) according to the manufacturer’s procedure. Following, insulin levels were normalized to pancreatic mass.

### Statistics

Unless stated otherwise, data presented are shown as mean ± standard error of the mean (SEM). Statistical analyses were performed using GraphPad Prism version 10.6.1 (GraphPad Software, San Diego, California, USA). For baseline characterization, one-way ANOVA was used to assess OCN effects for each sex (genotype effect). In longitudinal body mass studies (genotype × time) and diet experiments (genotype × diet), two-way ANOVA was consistently applied within each sex. In addition to analyzing individual effects of variables like genotype, diet, and time, their interactions were also examined. Post hoc comparisons with Bonferroni correction were conducted to identify specific group differences. In addition, Pearson correlation analysis and non-linear regression analysis were applied as appropriate. For non-linear regression analysis, outliers detected by the ROUT method^67^ were excluded from the calculation but are displayed in the figures as red stars. A *P*-value of < 0.05 was considered statistically significant for all tests.

Data corresponding to the images presented in the manuscript, as well as further data, are available in the supplemental tables. Additionally, raw data of the high-fat/low-fat diet study are deposited in Mendeley Data (10.17632/tksys9xtgs.1).^63^

## Supplementary information

Supplement

## Data Availability

The data that support the findings of this study are available from the corresponding author upon reasonable request. In addition, raw data of the high-fat/low-fat diet study are deposited in Mendeley Data (10.17632/tksys9xtgs.1).
