# Supplementary material for: Osteocalcin-dependent and -independent metabolic dysregulation in a mouse model of Osteogenesis imperfecta
Source: Bone Res. 2026 Jul 16;14:74. doi: 10.1038/s41413-026-00553-1 (PMC13376579; doi:10.1038/s41413-026-00553-1)
Supplement: Supplementary file 1 — Supplement [file 41413_2026_553_MOESM1_ESM.pdf]

Supplemental Material for the manuscript ***Osteocalcin-dependent and -independent metabolic dysregulation in a mouse model of osteogenesis imperfecta by*** Josephine T. Tauer, Frank Rauch, Mathieu Ferron, Svetlana V. Komarova

Supplemental Figures

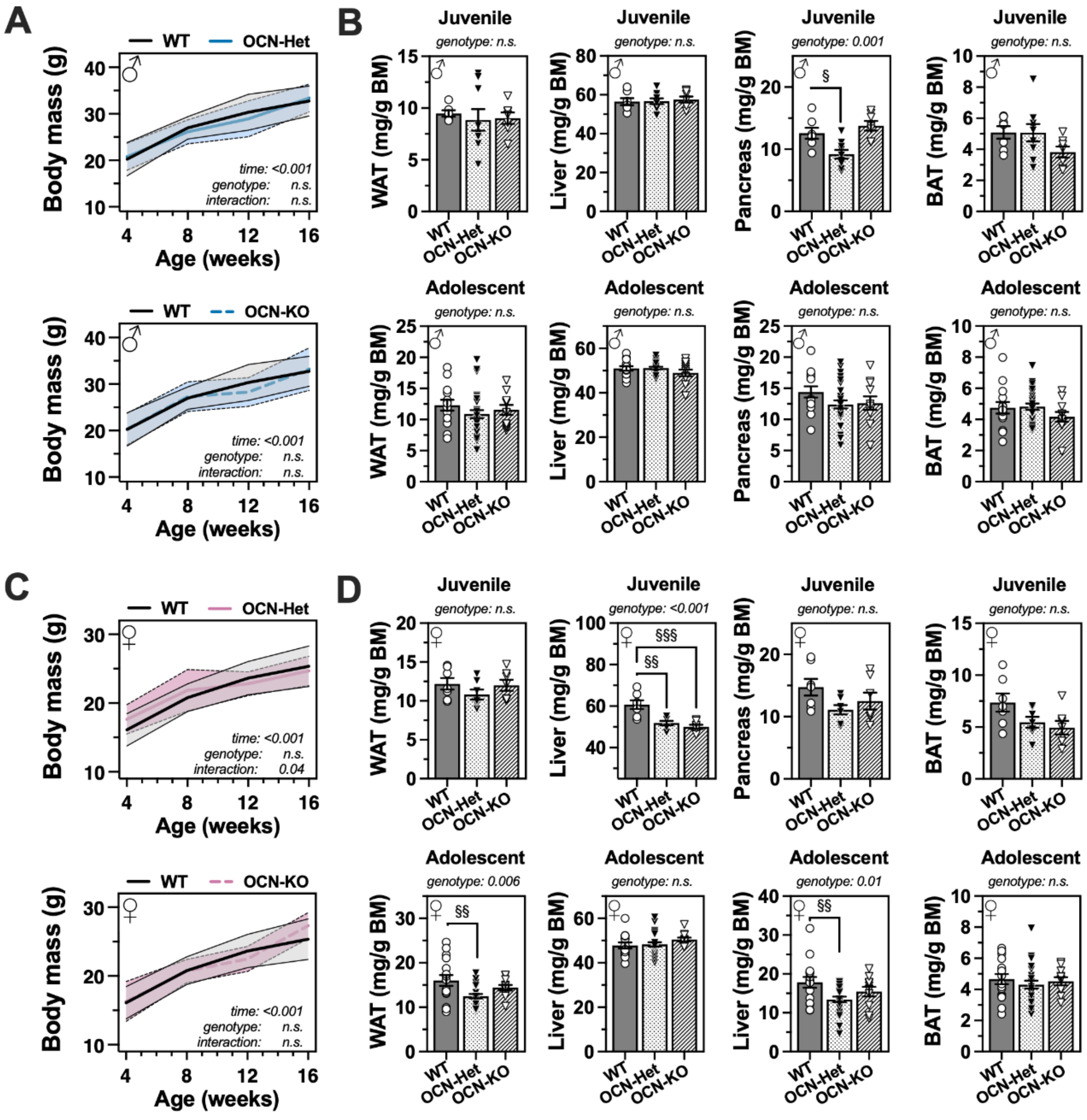

Supplemental Figure S1: Body mass (BM) development of WT and OCN-deficient mice on regular chow. Data are mean±SD (A,C) or mean±SEM and individual measurements per genotype (B,D). Statistical analysis: (A,C) two-way ANOVA to assess overall effect of genotype, time, and interaction, followed by Bonferroni post-test to assess genotype effect per age: * $p<0.05$, ** $p<0.01$, *** $p<0.001$. (B,D) one-way ANOVA within each sex to assess for overall genotype effect, followed by Bonferroni post-test to assess OCN effect by comparison to WT only: § $p<0.05$, §§ $p<0.01$, §§§ $p<0.001$.

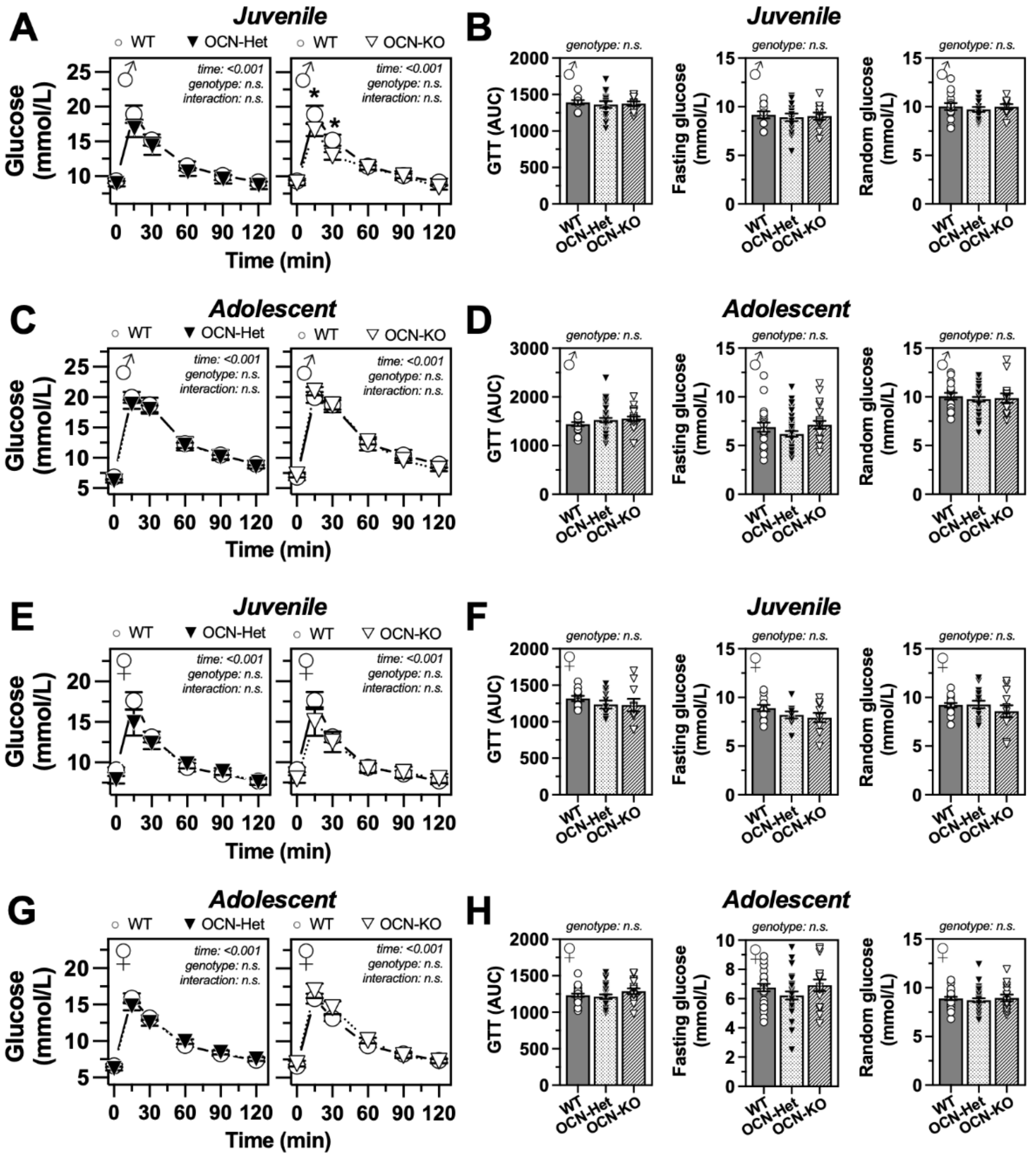

Supplemental Figure S2: Glucose metabolism in male and female WT and OCN-deficient mice on regular chow at juvenile and adolescent age. Juvenile, 4 weeks of age. Adolescent, 8-12 weeks of age. (A, B, C, D) Glucose tolerance tests (GTT) over time. (B, D, F, H) Left panel: Area under the curve (AUC) analysis of performed GTT. Middle panel: Fasting glucose levels (mmol/L) at time point 0 of the GTT. Right panel: Random blood glucose levels (mmol/L). Data are means ± SEM, and individual measurements per genotype. Statistical analysis: (A, B, C, D) two-way ANOVA of repeated measures assessing the overall effect of time, genotype, and their interaction, followed by Bonferroni post-test to assess significant difference per time point: * $p<0.05$. (B, D, F, H) One-way ANOVA to assess for overall genotype effect, followed by Bonferroni post-test to assess OCN effect by comparison to WT only: n.s.

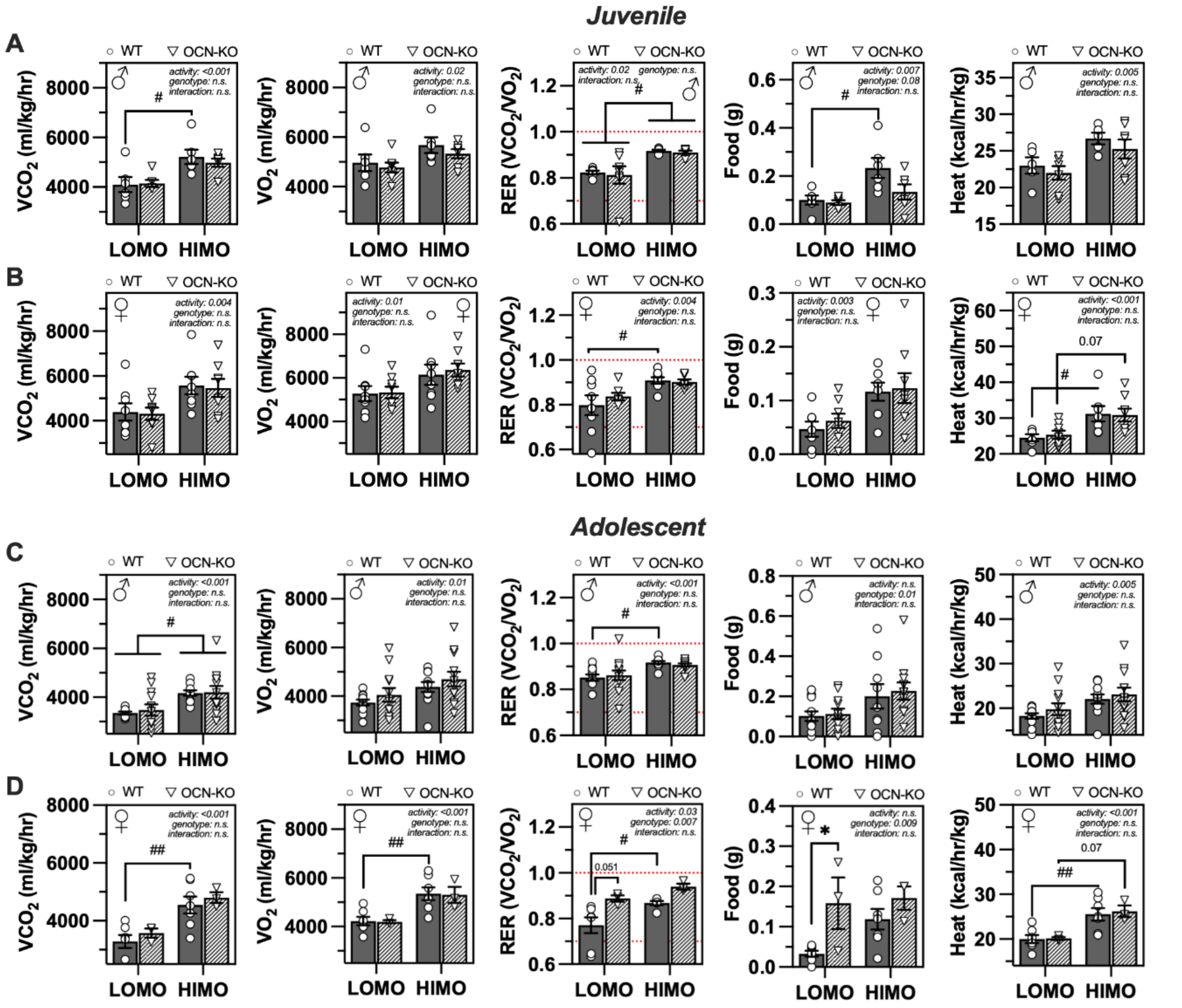

Supplemental Figure S3: Metabolic phenotype in regular chow-fed male (A, C) and female (B;D) WT and OCN-deficient mice at juvenile and adolescent age. Juvenile, 4 weeks of age. Adolescent, 8-12 weeks of age. LOMO, low mobility; HIMO, high mobility; RER, respiratory exchange ratio.Data are presented as mean±SEM and individual measurements. Statistical analysis: two-way ANOVA within each sex evaluating the overall effects of genotype, mobility, and their interaction, followed by Bonferroni post-hoc testing for genotype effects: * p<0.05, ** p<0.01, *** p<0.001; or assessing mobility effects: # p<0.05, ## p<0.01, ### p<0.001.

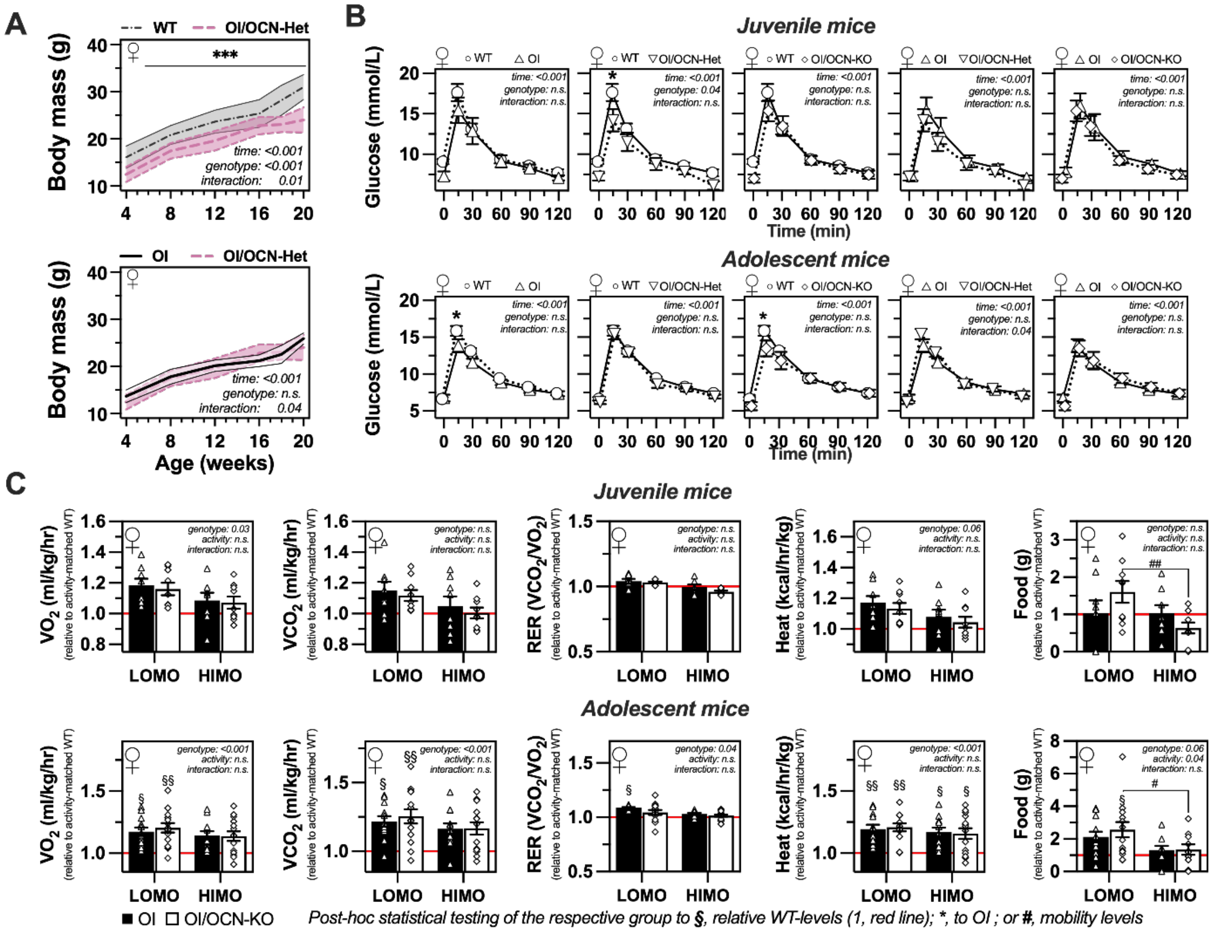

Supplemental Figure S4: Body mass development (A), glucose tolerance testing (B) and metabolic phenotype (C) in regular chow-fed female WT, OI and OI/OCN-KO mice at juvenile and adolescent age. LOMO, low mobility; HIMO, high mobility; RER, respiratory exchange ratio. (C,D) Measurements are normalized to age-, sex- and mobility-matched WT mice, and are presented as a fold-change from WT (set to 1, red line). Data are presented as mean±SD (A) or mean±SEM (B,C) and individual measurements (D). Statistical analysis: (A,B) two-way ANOVA of repeated measures assessing the overall effect of time, genotype, and their interaction, followed by Bonferroni post-test to assess significant difference per age (A) or time point (B): diet effect * $p<0.05$, ** $p<0.01$, *** $p<0.001$. (C) two-way ANOVA within each sex evaluating the overall effects of genotype, mobility, and their interaction, followed by Bonferroni post-hoc testing for genotype effects by comparison to mobility-matched OI: * $p<0.05$, ** $p<0.01$, *** $p<0.001$; or comparison to WT: § $p<0.05$, §§ $p<0.01$, §§§ $p<0.001$, or assessing mobility effects (# $p<0.05$, ## $p<0.01$, ### $p<0.001$).

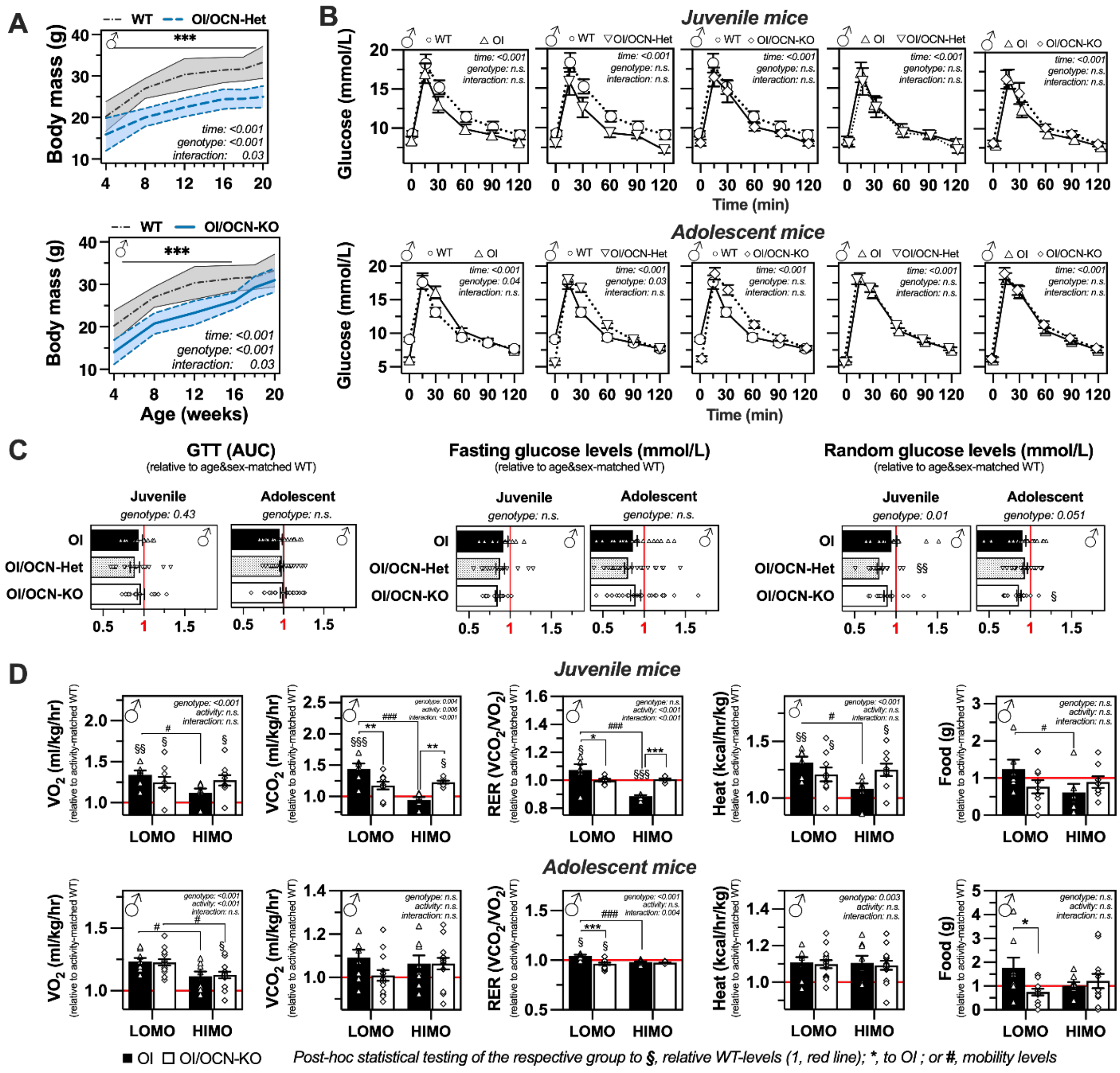

Supplemental Figure S5: Body mass development (A), glucose homeostasis testing (B,C) and metabolic phenotype (D) in regular chow-fed male WT, OI and OI/OCN-KO mice at juvenile and adolescent age. LOMO, low mobility; HIMO, high mobility; RER, respiratory exchange ratio. (C,D) Measurements are normalized to age-, sex- and mobility-matched WT mice, and are presented as a fold-change from WT (set to 1, red line). (C) Area under the curve (AUC) for glucose tolerance tests (GTT) shown in B. Fasting glucose levels (mmol/L) at time point 0 of the GTT. Random glucose levels (mmol/L). Absolute measurement values are listed in **Supplemental Table S8-S9.** Data are presented as mean±SD (A) or mean±SEM (B,C,D) and individual measurements (C,D). Statistical analysis: (A,B) two-way ANOVA of repeated measures assessing the overall effect of time, genotype, and interaction, followed by Bonferroni post-test to assess significant difference per age (A) or time point (B): * p<0.05, ** p<0.01, *** p<0.001. (C) one-way ANOVA to assess for overall genotype effect, followed by Bonferroni post-test to assess OCN effect by comparison to OI: n.s. or comparison of OCN-levels: n.s.; or assess OI and OCN effect by comparison to WT only: § p<0.05, §§ p<0.01. (D) two-way ANOVA within each sex evaluating the overall effects of genotype, mobility, and their interaction, followed by Bonferroni post-hoc testing for genotype effects by comparison to mobility-matched OI mice: * p<0.05, ** p<0.01, *** p<0.001; or comparison to WT mice: § p<0.05, §§ p<0.01, §§§ p<0.001, or assessing mobility effects (# p<0.05, ## p<0.01, ### p<0.001).

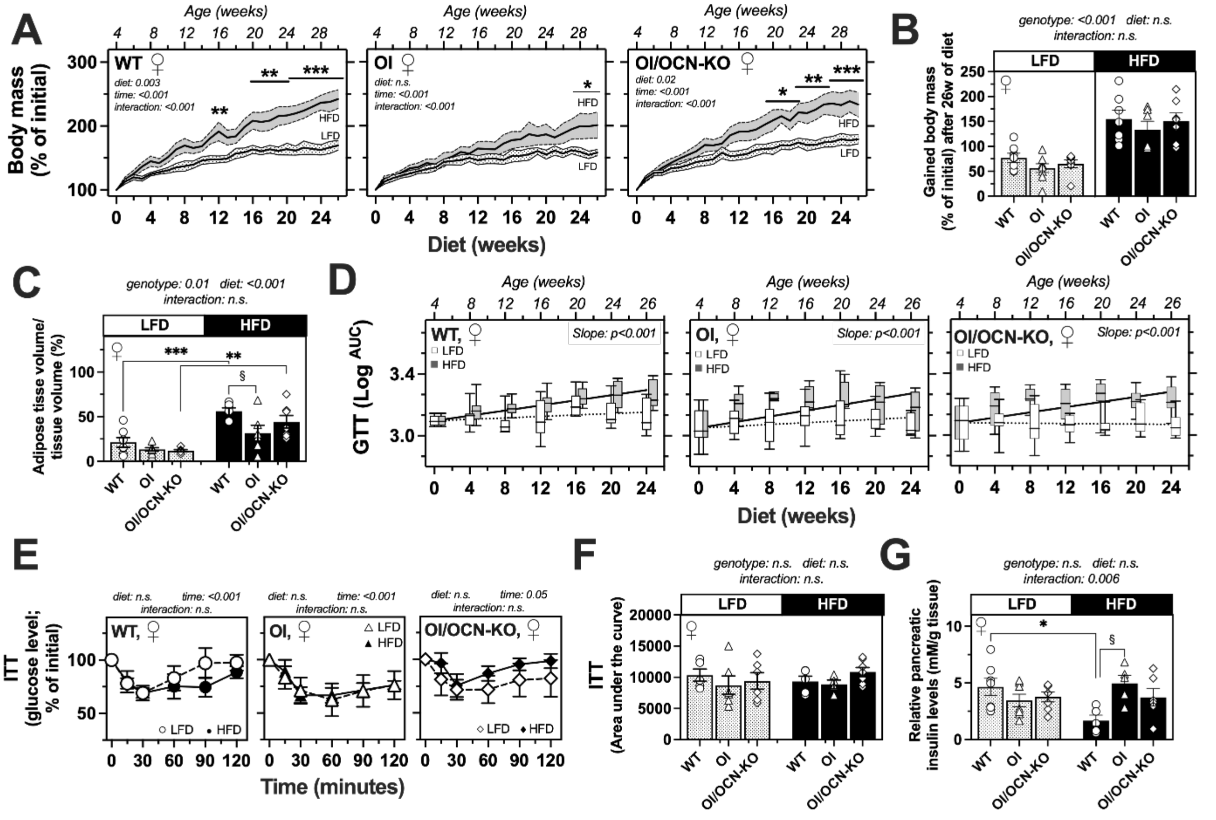

*(B, C, F, G) Post-hoc statistical testing of the respective group to §, WT; *, diet ; or #, OCN-genotype*

Supplemental Figure S6: Obesity development (A-C), glucose (D) and insulin (E-G) metabolism in low-fat (LFD) or high-fat diet (HFD) fed female WT, OI and OCN-KO mice. (A) Body mass progression as % change from the start of dietary intervention during the 26 weeks on LFD or HFD. Data are mean±SD (B) Gained body mass after 4 or 26 weeks of LFD or HFD. Data are mean±SEM and individual measurements per genotype. (C) Adipose tissue volume (AV) over tissue volume (TV) after 26 weeks of LFD or HFD. Data are mean±SEM and individual measurements per genotype. (D) Glucose tolerance test (GTT) was performed before (=0) and at 4, 8, 12, 16, 20 and 24 weeks on LFD or HFD; area under the curve (AUC) was quantified and log10 transformed. Data are mean±SEM per time point. (E) Insulin tolerance tests (ITT) after 24 weeks of diets, shown as changes in glucose levels (% from initial glucose levels) after insulin injection at time point 0. Data are mean±SEM and individual measurements per genotype. (F) Average AUC of ITT depicted in E. Data are mean±SEM and individual measurements per genotype. (G) Relative pancreatic insulin levels normalized to pancreatic tissue weights. . Statistical analysis: (A,E) Two-way ANOVA of repeated measures assessing the overall effect of diet, time, and their interaction, followed by Bonferroni post-test to assess diet effect (* p<0.05, ** p<0.01, *** p<0.001). (B) Two-way ANOVA assessing the overall effect of genotype, time, and their interaction, followed by Bonferroni post-test to assess genotype effect within each diet (n.s.) (C,F,G) Two-way ANOVA assessing the overall effect of genotype, diet, and their interaction, followed by Bonferroni post-test to assess diet effect (* p<0.05, ** p<0.01, *** p<0.001) and to assess genotype effect by comparison to diet-matched OI: n.s. or diet-matched WT: § p<0.01 . (D) Non-linear regression analysis with evaluation of statistical differences between curve slopes.

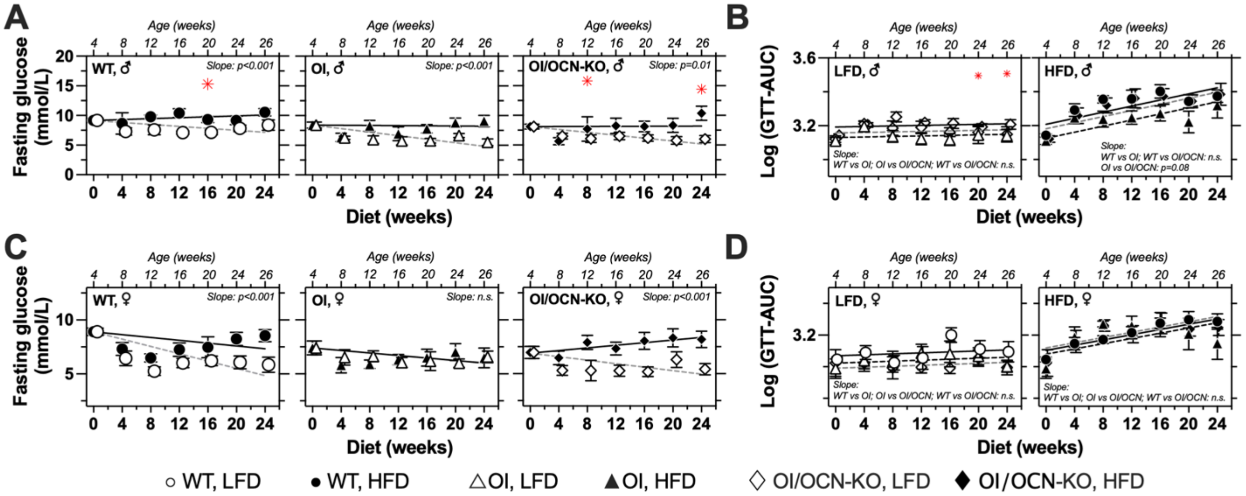

Supplemental Figure S7: Fasting glucose (A,C) and glucose tolerance testing (GTT) (B,D) in low-fat (LFD) or high-fat diet (HFD) fed male (A,B) and female (C,D) WT, OI and OCN-KO mice. Fasted glucose levels represent time point 0 of glucose tolerance testing. Data are presented as mean±SEM. Statistical analysis: Non-linear regression analysis over time with evaluation of statistical differences between curve slopes. Red stars indicate outliers that have been identified using ROUT method (see Material/Methods for details) and are shown for completeness of the dataset.

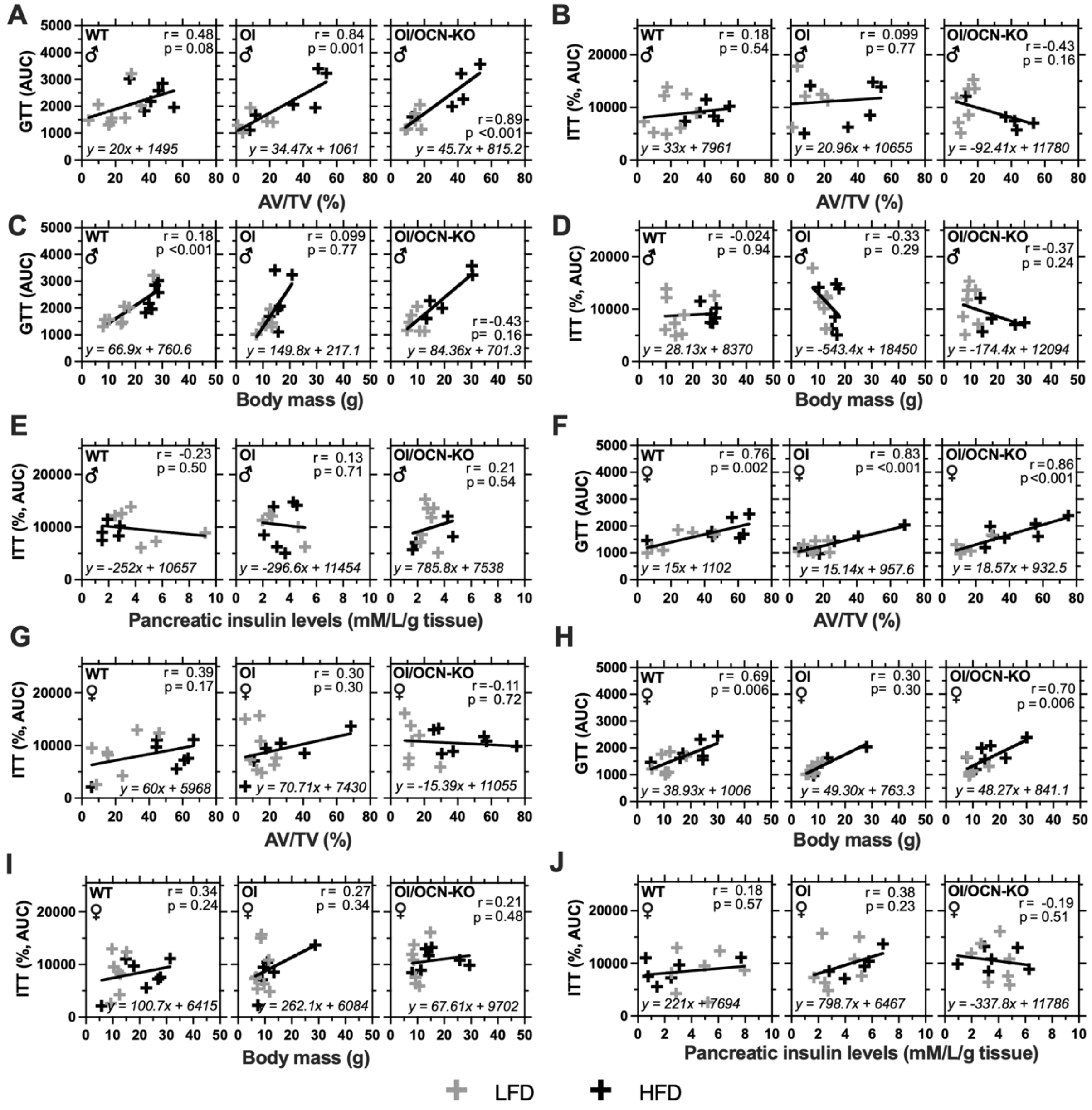

Supplemental Figure S8: Correlation analysis of glucose and insulin homeostasis to body mass, adiposity, or pancreatic insulin levels of low-fat (LFD) or high-fat diet (HFD) fed WT, OI and OCN-KO mice. Statistics: Pearson correlation analysis of individual mice after 26 weeks of diet intervention with LFD and HFD. AV/TV, adipose tissue volume / tissue volume. ITT, insulin tolerance testing. AUC, area under the curve.

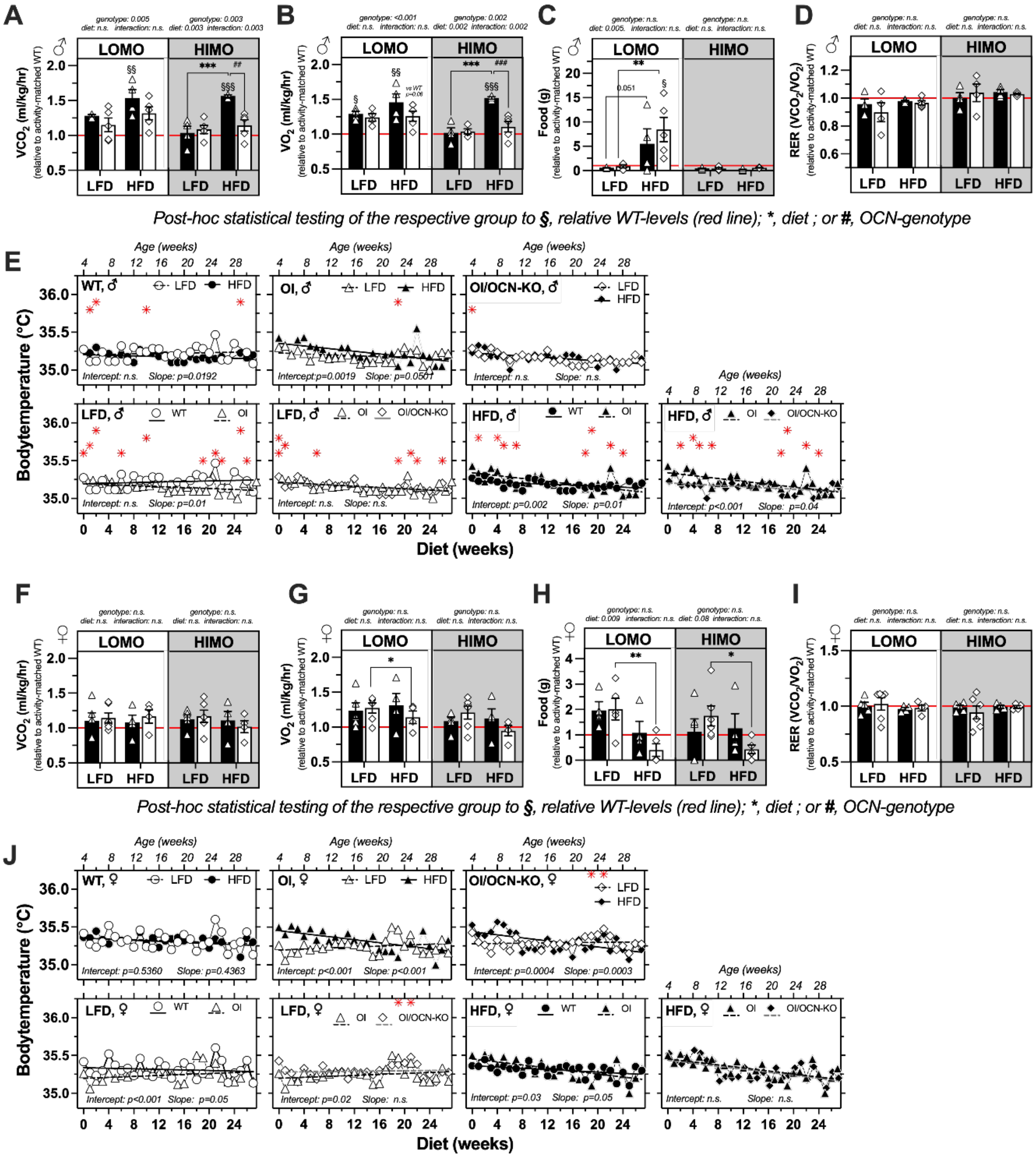

Supplemental Figure S9: Indirect calorimetry assessment and body surface temperature of male (A-E) and female (F-J) of low-fat (LFD) or high-fat diet (HFD) fed OI and OI/OCN-KO mice. Data are mean±SEM and individual measurements. LOMO, low mobility levels. HIMO, high mobility levels. RER, respiratory exchange ratio. Indirect calorimetry parameters: (A,F) relative carbon dioxide production, (B,G) relative oxygen consumption, (C,H) relative food intake, (D,I) relative RER. Statistics: (A-D) two-way ANOVA within each sex and mobility to assess the overall effect of OCN levels (a), the overall diet effect (b), and the interaction of diet and OCN levels (c); followed by Bonferroni post-hoc testing for diet effect: * p<0.05, ** p< 0.01, *** p<0.001; and genotype effect by comparison to WT: § p<0.05, §§ p<0.01, or to OI: #, p<0.05. (E) Non-linear regression analysis over a 26-week diet intervention. Red stars indicate outliers that have been identified using ROUT method (see Material/Methods for details) and are shown for completeness of the dataset.

Supplemental Material for the manuscript ***Osteocalcin-dependent and -independent metabolic dysregulation in a mouse model of osteogenesis imperfecta by*** Josephine T. Tauer, Frank Rauch, Mathieu Ferron, Svetlana V. Komarova

Supplemental Tables

Supplemental Table S1: Overview of genotype distribution per generation.

Generation	Sex	N	WT	OI	OI/OCN-Het	OI/OCN-KO	FD
F1	♂	176	13.6%	14.2%	26.1%	-	2%
	♀	146	13.0%	13.0%	39.7%	-	
F2 - F10	♂	219	9.6%	13.7%	24.7%	18.3%	10%
	♀	228	6.6%	18.4%	28.5%	15.8%	

F1 generation was derived from the crossing of *Col1a1*$^{Jrt/+}$ (OI) with *Bglap*$^{+/-}$ (OCN); F2 – F10 generation was derived from the crossing of OI/OCN-Het with OI/OCN-Het of the previous generations. FD, found dead. Genotype details: WT: *Col1a1*$^{+/+}$/*Bglap*$^{+/+}$; OI: *Col1a1*$^{Jrt/+}$/*Bglap*$^{+/+}$; OI/OCN-Het: *Col1a1*$^{Jrt/+}$/*Bglap*$^{+/-}$; OI/OCN-KO: *Col1a1*$^{Jrt/+}$/*Bglap*$^{-/-}$.

Supplemental Table S2: Mouse serum levels of tOCN, GLA13-OCN, and GLU-OCN at 4 weeks of age.

Generation	Statistics (genotype effect)	WT	OCN-Het	OI	OI/OCN-Het
Male mice		N=7	N=5	N=4	N=4-5
tOCN (ng/mL)	<0.001	538.5±15.0	284.5±24.0 §§§	728.7±85.6 §§	296.9±12.4 *** §§
GLU13-OCN (ng/mL)	<0.001	85.3±6.15	26.1±7.20 §§	159.2±22.5 §§§	40.6±6.01*** §
GLA-OCN (ng/mL)	<0.001	453.2±13.5	258.3±17.7 §§§	569.6±64.5 §	259.6±12.1 *** §§§
Female mice		N=5-6	N=5	N=6	N=6
tOCN (ng/mL)	0.003	472.9±93.7	351.0±33.2	666.1±65.9 (vs WT p=0.08)	302.7±33.1 **
GLU13-OCN (ng/mL)	0.001	92.6±27.2	62.0±10.5 (vs WT p=0.06)	154.7±27.6 §	31.6±8.43 ***
GLA-OCN (ng/mL)	0.001	395.1±67.4	289.0±23.0	511.3±43.2 §	271.2±26.4 **

Data are means±SEM. Statistical analysis: one-way ANOVA within each sex to assess for overall genotype effect followed by Bonferroni post-test to assess OCN effect by comparison to WT only: § p<0.05, §§ p<0.01, §§§ p<0.001, and to OI only: * p<0.05, ** p<0.01, *** p<0.001.

Supplemental Table S3: Fur phenotype distribution per generation.

Generation [$]	Fur color	N	WT	OCN-Het	OCN-KO	OI	OI/OCN-Het	OI/OCN-KO
F1	White	-	-	-	-	-	-	-
	Black	-	-	-	-	-	-	-
	Grey	328	13.1%	39.9%	-	13.4%	31.7%	-
F2 - F10	White	107	11.2%	18.7%	16.8%	14.9%	24.3%	14.9%
	Black	113	8.8%	23.0%	9.7%	19.5%	23.9%	15.0%
	Grey	222	6.3%	21.2%	9.9%	15.3%	27.9%	19.4%

§, 1st generation derived from the crossing of *Col1a1*$^{Jrt/+}$ with *Bglap*$^{-/-}$; 2nd – 10th generation derived from the crossing of OI/OCN-Het with OI/OCN-Het of the previous generation, respectively. White fur, related to FVB background; Black fur, related to C57BL6 background; Grey fur, crossbreeding of both strains.

Supplemental Table S4: Overview of breeding efficiency per genotype and sex on mixed background.

Genotype	WT		OI		OI/OCN-Het		OI/OCN-KO	
Sex	Male	Female	Male	Female	Male	Female	Male	Female
**Total number of animals used for breeding**	9	17	18	33	67	109	7	27
**Number of successful breeding pairs**	9 (100%)	17 (100%)	13 (72%)	28 (85%)	48 (72%)	93 (85%)	17 (100%)	22 (81%)
**Number of non-successful breeding pairs**	-	-	5 (28%)	5 (15%)	19 (28%)	16 (15%)	-	5 (19%)
**Number of pups born**	123	143	237	217	805	794	75	120
**Number of pups died post-birth**	2 (1.6%)	0 (0%)	10 (4.2%)	11 (5.1%)	69 (8.6%)	82 (10.3%)	5 (6.7%)	7 (5.8%)
**Mean litter size (min; max)**	7 (4; 11)	8 (6; 11)	7 (1; 12)	7 (1; 12)	6 (1; 11)	5 (1; 11)	4 (1; 8)	5 (2; 9)

Supplemental Table S5: Body mass development of male and female mice on regular chow.

	Genotype							
	WT		OI		OI/OCN-Het		OI/OCN-KO	
**Age (weeks)**	**Mean±SD**	**N**	**Mean±SD**	**N**	**Mean±SD**	**N**	**Mean±SD**	**N**
Male mice								
**4**	20.25±3.58	20	15.27±2.51	19	15.94±3.94	34	14.05±2.89	26
**8**	26.99±2.41	25	20.42±1.84	20	20.04±2.2	33	20.78±2.46	16
**12**	30.35±3.86	25	22.77±2.42	24	22.47±2.24	29	23.29±2.81	32
**16**	31.45±3.06	23	24.38±1.49	16	24.45±2.38	29	26.12±1.92	17
**18**	31.69±2.89	7	25.04±1.4	10	24.51±2.18	15	29.26±2.6	5
**20**	33.27±3.85	14	26.36±2.75	9	25.01±2.58	20	30.98±2.82	6
Female mice								
**4**	16.09±2.36	17	13.64±1.35	21	12.46±1.62	20	13.12±2.32	18
**8**	20.78±2.02	21	17.78±1.59	12	17.44±1.64	29	16.76±1.83	16
**12**	23.64±2.44	16	20.13±1.21	22	19.58±2.08	33	19.55±2.2	12
**16**	25.35±2.95	11	21.2±1.24	9	22.79±1.85	23	22.8±1.58	10
**18**	28.16±3.17	8	22.53±1.92	4	23.03±1.59	13	23.83±1.93	3
**20**	30.98±2.63	6	25.9±1.17	4	23.99±2.64	31	24.92±2.81	5

Supplemental Table S6: Relative organ weights (mg/g body mass) of juvenile (4 weeks of age) and adolescent mice (8 and 12 weeks of age) fed a regular chow.

Organ	Statistics (genotype effect)	WT	OI	OI/OCN-Het	OI/OCN-KO
Juvenile male mice		N=6-7	N=6	N=9	N=5
**Liver**	<0.001	56.5±1.8	49.6±2.2 §	58.6±1.3 **	63.5±1.7 ***
**Pancreas**	0.16	12.6±0.9	10.1±1.3	10.9±0.6	9.8±0.6
**Brown adipose tissue**	0.23	5.1±0.4	4.3±0.5	4.3±0.5	5.8±0.6
**White adipose tissue**	<0.001	9.5±0.3	5.2±0.6 §§§	6.6±0.5 §§	8.0±0.6 **
Juvenile female mice		N=6	N=6	N=8	N=4
**Liver**	0.02	60.7±2.1	52.8±1.2	52.2±2.6 (vs. WT: p=0.051)	59.6±2.0
**Pancreas**	0.004	15.4±1.4	11.0±0.9 §	10.6±0.6 §§	10.0±1.1 §
**Brown adipose tissue**	0.005	7.4±0.9	4.9±0.4	3.9±0.3 §§	5.1±0.7
**White adipose tissue**	0.008	12.2±0.7	7.0±1.0 §§	8.9±0.7	10.4±2.1
Adolescent male mice		N=14	N=12	N=15	N=9
**Liver**	0.04	51.0±1.0	56.2±0.9 §	53.8±1.4	53.9±1.5
**Pancreas**	0.69	14.4±0.9	13.5±1.2	12.6±0.9	13.2±1.6
**Brown adipose tissue**	0.13	4.8±0.4	4.2±0.2	3.8±0.3	3.7±0.3
**White adipose tissue**	0.03	12.3±0.9	9.8±0.5	9.5±0.7 §	11.3±0.9
Adolescent female mice		N=15	N=7	N=15	N=5
**Liver**	0.24	47.8±1.4	51.1±1.7	52.1±1.8	51.5±3.2
**Pancreas**	0.01	17.8±1.4	18.7±1.5	12.9±0.9 * §	14.8±1.8
**Brown adipose tissue**	0.98	4.7±0.3	4.6±0.6	4.5±0.4	4.4±0.4
**White adipose tissue**	0.17	16.0±1.2	13.2±2.2	12.5±0.8	13.0±2.0

Data are means±SEM. Interscapular brown adipose tissue and inguinal white adipose tissue were analysed at the described age groups. Statistical analysis: one-way ANOVA within each sex to assess for overall genotype effect followed by Bonferroni post-test to assess OCN effect by comparison to OI only: * p<0.05, ** p<0.01, *** p<0.001; and assess OI and OCN effect by comparison to WT only: § p<0.05, §§ p<0.01, §§§ p<0.001.

Supplemental Table S7: Femoral structural and mechanical parameters at adolescent age.

Parameter	Statistics (genotype effect)	WT	OI	OI/OCN-Het	OI/OCN-KO
Bone structural parameter		N=17	N=13	N=23	N=7
**Bone length (mm)**	<0.001	15.1±0.1	14.1±0.1 §§§	14.1±0.08 §§§	14.1±0.1 §§§
**BV/TV (%)**	<0.001	16.1±1.5	5.3±0.6 §§§	7.5±0.5 §§§	5.7±0.2 §§§
***v*BMD (gHA/cc)**	<0.001	0.21±0.02	0.08±0.01 §§§	0.09±0.01 §§§	0.07±0.01 §§§
**Tb.N. (1/mm)**	<0.001	3.39±0.24	1.42±0.14 §§§	2.01±0.13 §§§	1.44±0.10 §§§
**Tb.Th. (um)**	<0.001	47.1±0.9	37.5±1.1 §§§	38.6±0.7 §§§	40.5±1.9 §§§
**Cortical Thickness (mm)**	<0.001	0.19±0.004	0.15±0.004 §§§	0.16±0.004 §§§	0.16±0.004 §§§
Bone mechanical parameter		N=14	N=11	N=14	N=7
**Maximal load (N)**	<0.001	12.6±0.5	5.67±0.44 §§§	6.44±0.36 §§§	5.18±0.49 §§§
**Energy until fracture (mJ)**	<0.001	10.0±1.32	0.40±0.10 §§§	0.57±0.09 §§§	0.49±0.14 §§§
**Stiffness (N/mm)**	<0.001	76.2±2.48	47.3±5.41 §§§	50.6±4.20 §§§	43.7±2.92 §§§

Data are means±SEM. BV/TV, bone volume per tissue volume, Tb.N., trabecular number; Tb.Th., trabecular thickness. Statistical analysis: one-way ANOVA within each sex to assess for overall genotype effect followed by Bonferroni post-test to assess OCN effect by comparison to OI only: n.s.; and assess OI and OCN effect by comparison to WT only: §§§ $p<0.001$.

Supplemental Table S8: Glucose tolerance testing (GTT) and fasting glucose levels (=T0min) of juvenile and adolescent male and female mice on regular chow. Absolute values are shown. Statistics are indicated in the respective figure.

	Genotype			
Juvenile male mice	**WT**	**OI**	**OI/OCN-Het**	**OI/OCN-KO**
	**N=9**	**N=10**	**N=15**	**N=16**
**Time (min)**	**Mean±SEM**	**Mean±SEM**	**Mean±SEM**	**Mean±SEM**
0	9.26±0.38	8.4±0.5	8.04±0.48	8.1±0.4
15	18.24±1.19	17±1.26	15.84±1.7	16.48±1.22
30	15.21±0.9	12.99±1.01	12.54±1.24	14.64±1.4
60	11.44±0.67	9.88±0.61	9.34±0.57	10.11±0.5
90	10.08±0.73	9.13±0.59	8.99±0.35	9.32±0.4
120	9.12±0.58	8.18±0.4	7.18±0.38	8±0.31
Juvenile female mice				
	**N=11**	**N=10**	**N=15**	**N=14**
**Time (min)**	**Mean±SEM**	**Mean±SEM**	**Mean±SEM**	**Mean±SEM**
0	9.08±0.31	7.3±0.57	7.2±0.46	6.98±0.51
15	17.61±1.04	15.43±1.57	14.18±1.36	15.35±1.26
30	13.13±0.66	12.85±1.62	11.5±1.13	13.47±1.22
60	9.38±0.51	9.13±0.75	8.76±0.73	9.21±0.57
90	8.56±0.39	8.28±0.46	7.8±0.34	8.17±0.52
120	7.7±0.43	7.09±0.3	6.14±0.52	7.45±0.51
Adolescent male mice				
	**N=19**	**N=23**	**N=32**	**N=25**
**Time (min)**	**Mean±SEM**	**Mean±SEM**	**Mean±SEM**	**Mean±SEM**
0	6.84±0.5	6.08±0.36	5.55±0.31	6.17±0.42
15	19.95±0.9	18.12±0.87	17.98±0.68	18.87±0.89
30	18.69±1.2	16.1±0.9	16.73±0.58	16.45±0.74
60	12.34±1.1	10.37±0.38	11.12±0.35	11.27±0.52
90	10.44±0.86	8.8±0.44	8.99±0.31	9.26±0.42
120	8.94±0.58	7.5±0.4	7.7±0.27	7.79±0.33
Adolescent female mice				
**Genotype**	**N=26**	**N=15**	**N=28**	**N=1)**
**Time (min)**	**Mean±SEM**	**Mean±SEM**	**Mean±SEM**	**Mean±SEM**
0	6.6±0.25	6.74±0.44	6.16±0.26	5.62±0.55
15	15.86±0.66	13.84±0.89	15.39±0.72	13.46±1.06
30	13.15±0.4	11.57±0.5	12.88±0.58	11.8±1.18
60	9.4±0.24	8.86±0.36	8.59±0.46	9.42±0.66
90	8.25±0.28	7.87±0.45	7.94±0.32	8.23±0.56
120	7.38±0.26	7.32±0.34	6.92±0.26	7.38±0.48

Supplemental Table S9: Random glucose levels of juvenile and adolescent male and female mice on regular chow. Absolute values are shown. Statistics are indicated in the respective figure.

	Juvenile mice				Adolescent mice			
**Sex**	**Male**		**Female**		**Male**		**Female**	
**Genotype**	**Mean±SEM**	**N**	**Mean±SEM**	**N**	**Mean±SEM**	**N**	**Mean±SEM**	**N**
**WT**	10.03±0.35	15	9.26±0.32	19	10.07±0.33	18	9.07±0.2	34
**OI**	9.48±0.62	15	7.56±0.34	12	9.09±0.42	23	10.05±1.2	13
**OI/OCN-Het**	8.64±0.42	23	7.68±0.32	21	9.43±0.27	27	8.71±0.42	29
**OI/OCN-KO**	9.02±0.45	15	8.73±1.22	8	9.8±0.81	21	9.75±0.85	8

Supplemental Table S10: Indirect calorimetric assessment of juvenile and adolescent male and female mice on regular chow. Absolute values are shown. Statistics are indicated in the respective figure.

Juvenile male mice						
**Genotype**	**WT (N=6)**		**OI (N=6)**		**OI/OCN-KO (N=8)**	
**Diet**	**LOMO**	**HIMO**	**LOMO**	**HIMO**	**LOMO**	**HIMO**
	**Mean±SEM**	**Mean±SEM**	**Mean±SEM**	**Mean±SEM**	**Mean±SEM**	**Mean±SEM**
**VCO_2 (ml/kg/hr)**	4098±301.5	5150±292.4	5897±354.5	4868±230.4	4817±251.3	6335±126.1
**VO_2 (ml/kg/hr)**	4963±329.4	5467±121	6649±274.4	6133±276.5	6198±330	6962±347.6
**RER (VCO_2/VO_2)**	0.82±0.007	0.9±0.008	0.88±0.035	0.79±0.007	0.82±0.008	0.9±0.006
**Energy expenditure (kcal/kg/hr)**	24.21±1.51	26.95±0.51	31.84±1.24	29.13±1.28	29.28±1.47	33.66±1.46
**Food (g)**	0.1±0.018	0.19±0.035	0.12±0.025	0.11±0.045	0.07±0.017	0.17±0.03
Juvenile female mice						
**Genotype**	**WT (N=8)**		**OI (N=8)**		**OI/OCN-KO (N=9)**	
**Diet**	**LOMO**	**HIMO**	**LOMO**	**HIMO**	**LOMO**	**HIMO**
	**Mean±SEM**	**Mean±SEM**	**Mean±SEM**	**Mean±SEM**	**Mean±SEM**	**Mean±SEM**
**VCO_2 (ml/kg/hr)**	4391±388.6	5566±389.6	5050±249.1	5839±349.9	4908±157	5589±195
**VO_2 (ml/kg/hr)**	5275±343.8	6142±465.8	6244±226.2	6671±306.9	6118±214.7	6582±242.6
**RER (VCO_2/VO_2)**	0.8±0.044	0.91±0.014	0.83±0.015	0.9±0.017	0.82±0.005	0.87±0.008
**Energy expenditure (kcal/kg/hr)**	25.44±1.93	30.29±2.06	29.81±1.08	32.67±1.41	28.82±0.91	31.62±1.06
**Food (g)**	0.05±0.014	0.11±0.016	0.05±0.018	0.12±0.025	0.08±0.015	0.07±0.016
Adolescent male mice						
**Genotype**	**WT (N=6)**		**OI (N=6)**		**OI/OCN-KO (N=9)**	
**Diet**	**LOMO**	**HIMO**	**LOMO**	**HIMO**	**LOMO**	**HIMO**
	**Mean±SEM**	**Mean±SEM**	**Mean±SEM**	**Mean±SEM**	**Mean±SEM**	**Mean±SEM**
**VCO_2 (ml/kg/hr)**	3348±61.51	4165±112.3	3652±122	4429±156.7	3378±82.87	4428±111.6
**VO_2 (ml/kg/hr)**	3739±121.2	4381±206.8	4127±98.22	4887±165	4107±85.11	4933±115.8
**RER (VCO_2/VO_2)**	0.85±0.014	0.92±0.004	0.89±0.008	0.91±0.007	0.82±0.01	0.9±0.002
**Energy expenditure (kcal/kg/hr)**	18.25±0.6	22.04±1.03	20.2±0.54	24.37±0.85	20.03±0.4	24.06±0.54
**Food (g)**	0.1±0.024	0.2±0.061	0.18±0.044	0.2±0.032	0.07±0.014	0.24±0.06
Adolescent female mice						
**Genotype**	**WT (N=8)**		**OI (N=8)**		**OI/OCN-KO (N=9)**	
**Diet**	**LOMO**	**HIMO**	**LOMO**	**HIMO**	**LOMO**	**HIMO**
	**Mean±SEM**	**Mean±SEM**	**Mean±SEM**	**Mean±SEM**	**Mean±SEM**	**Mean±SEM**
**VCO_2 (ml/kg/hr)**	3275±223	4545±291.3	3989±124.6	5288±178.6	4107±168.5	5303±204.4
**VO_2 (ml/kg/hr)**	4209±171	5353±264.1	4941±141.7	6125±175.3	5072±159.1	6088±208.5
**RER (VCO_2/VO_2)**	0.77±0.035	0.87±0.009	0.84±0.005	0.9±0.007	0.81±0.018	0.88±0.01
**Energy expenditure (kcal/kg/hr)**	19.93±0.88	25.53±1.4	23.8±0.7	29.92±0.87	24.09±0.63	29.55±1.08
**Food (g)**	0.03±0.008	0.12±0.026	0.06±0.01	0.16±0.031	0.07±0.014	0.16±0.038

Supplemental Table S11: Body mass (% of body mass at start of diet) in male WT, OI, and OI/OCN-KO mice during dietary intervention.

Genotype	WT				OI				OI/OCN-KO			
Statistics:	diet: <0.001. time: <0.001. interaction: <0.001				diet: n.s.. time: <0.001. interaction: <0.001				diet: 0.007. time: <0.001. interaction: <0.001			
Weeks of diet	LFD		HFD		LFD		HFD		LFD		HFD	
	Mean±SEM	N	Mean±SEM	N	Mean±SEM	N	Mean±SEM	N	Mean±SEM	N	Mean±SEM	N
**0**	100±0	10	100±0	10	100±0	10	100±0	10	100±0	9	100±0	9
**1**	112.1±4.14	8	128.1±4.06	6	122.6±2.47	9	132.5±9.78	6	117.2±4.4	9	136±9.02	8
**2**	118.4±4.71	8	136.1±3.96	6	137.1±3.82	9	142±12.28	6	127.6±5.71	9	154.5±10.26	8
**3**	124±5.48	8	145.4±5.72	6	145.4±6.89	7	150.3±12.1	6	134±6.67	9	164.6±10.06	8
**4**	128.5±5.8	8	154.5±6.38	6	151.1±5.28	8	157.1±12.92	6	138.9±7.5	9	170±10.81	8
**5**	132.7±6.35	8	161.9±7.14	6	151.8±5.28	8	160.9±12.51	6	139.6±8.13	9	171.5±12.37	8
**6**	138.3±6.98	8	169.6±9.23	6	156.8±6.63	8	168.2±15.36	6	148.9±9.43	9	183.5±13.89	6
**7**	141.9±7.67	8	183.3±10.19 *	6	161.2±6.86	8	172.6±14.66	6	148.8±9.41	9	185.5±15.17	6
**8**	146.2±8.03	8	191.4±9.1 **	6	163.6±5.93	8	176.2±15.71	6	155.5±9.92	9	193.2±16.73	6
**9**	145.7±7.12	8	196.8±10.62 **	6	164.7±6.66	8	177.6±15.61	6	155.4±9.33	9	197±16.92	6
**10**	148.8±7.83	8	198.7±9.65 **	6	169.1±7.13	8	182.9±16.43	6	161.6±10.08	9	200±17.2	6
**11**	151.2±8.35	8	209.3±11.68 ***	6	166.7±8.18	8	189.6±16.44	6	160.3±11.47	9	208.6±18.74	6
**12**	153.5±7.75	8	217.2±12.17 ***	6	169.7±7	8	195.3±15.87	6	164.9±13.43	8	216.6±23.42	6
**13**	155.3±8.24	8	223.2±11.51 ***	6	173.9±8.2	8	197.3±15.42	6	153.5±9.89	7	219.8±22.83 *	6
**14**	159.5±9.3	8	225.7±12.11 ***	6	173.7±11.38	8	203.9±16.51	6	156.8±12.56	7	225.2±25.11 *	6
**15**	159.6±9.71	8	233.2±13.6 ***	6	172.2±8.74	8	207±16.73	6	157.8±11.71	7	231.8±26.45 **	6
**16**	162±9.81	8	237.4±12.94 ***	6	175.7±9.68	8	209±15.86	6	157.2±11.29	7	232.3±27.26 **	6
**17**	161.3±10.12	8	239.6±11.99 ***	6	178.3±8.25	8	210.1±17.69	6	159.2±12.68	7	237±27.53 **	6
**18**	156.5±7.98	8	243.6±12.13 ***	6	179.6±9.4	8	216.4±19.05	6	164.6±11.29	7	240.3±29.67 **	6
**19**	159±8	8	244.5±12.24 ***	6	181.7±9.25	8	220.6±17.99	6	161.1±10.58	7	220.3±19.92 **	6
**20**	160.6±8.09	8	246.8±11.58 ***	6	183.1±8.33	8	221.7±17.9	6	163.5±10.77	7	227.5±24.79 **	6
**21**	162.7±8.21	8	248.1±13.62 ***	6	184.2±10.02	8	222.3±16.95	6	162.5±9.5	7	230.5±27.97 **	6
**22**	163.7±9.14	8	247.1±14.48 ***	5	181.6±10.87	8	210.9±14.79	5	164.5±8.92	7	204.5±10.07 **	6
**23**	162.7±8.25	8	258.8±13.02 ***	6	184.3±9.5	8	225.1±16.25	6	164±8.46	7	234.3±24.34 **	6
**24**	165.1±8.81	8	258.7±11.76 ***	6	185.7±10.56	8	226.9±16.65	6	162.9±8.81	7	237±23.72 **	6
**25**	163.4±9.7	8	253.2±9.83 ***	6	187.5±12.58	8	227.6±15.89	6	167.3±9.28	7	234.9±20.58 **	6
**26**	164.8±6.98	8	259.5±9.33 ***	6	187.3±9.6	8	225.2±18.34	6	167.9±8.48	7	235±23.33 **	6

LFD, low-fat diet. HFD, high-fat diet. Statistics: two-way AOVA of repeated measurements assessing the overall effect of diet. time. and their interaction. followed by Bonferroni post-test to assess diet effect (* p<0.05. ** p<0.01. *** p<0.001).

Supplemental Table S12: Body mass (% of body mass at start of diet) in female WT, OI, and OI/OCN-KO mice during dietary intervention.

Genotype	WT				OI				OI/OCN-KO			
Statistics	diet: 0.003. time: <0.001. interaction: <0.001				diet: n.s.. time: <0.001. interaction: <0.001				diet: 0.02. time: <0.001. interaction: <0.001			
Weeks of diet	LFD		HFD		LFD		HFD		LFD		HFD	
	Mean±SEM	N	Mean±SEM	N	Mean±SEM	N	Mean±SEM	N	Mean±SEM	N	Mean±SEM	N
0	100±0	7	100±0	8	100±0	6	100±0	6	100±0	7	100±0	7
1	112.9±3.77	7	116.3±3.97	8	112.3±3.97	6	116.8±4.57	6	117.5±3.62	7	116.6±4.82	6
2	119.6±4.18	7	125±5.67	8	117.9±4.86	6	120.2±4.2	6	126.4±4.04	7	125.9±5.27	6
3	117.5±3.91	7	136.4±6.75	8	121.8±3.66	6	126.5±3.91	6	126.9±4.25	7	134.5±7.3	6
4	124.4±2.72	7	144.8±8.05	8	125.3±5.07	6	133.3±5.44	6	133.5±5.71	7	142±6.79	6
5	127.6±3.12	7	141.7±6.84	8	128.8±3.62	6	135.3±6.7	6	135±5.19	7	148.7±5.25	6
6	130.3±4.51	7	150.5±8.47	8	132.1±6.44	6	141.6±6.38	6	138.7±6.72	7	154.5±6.77	6
7	135.1±6.43	7	163.3±10.28	8	134.5±5.36	6	139.4±8.55	6	143.6±7.74	7	160.4±9.2	6
8	137.1±8.21	7	169.4±11.09	8	143.4±4.58	6	146.6±11.35	6	145.6±7.78	7	170.2±10.73	6
9	139.3±5.62	7	165.5±8.67	7	144.7±6.05	6	151.7±7.29	6	147.9±7.97	7	169.6±9.02	6
10	145.8±5.85	7	168.2±8.43	7	146.2±8.15	6	158.9±8.66	6	157.9±9.18	7	172.3±10.96	6
11	147.6±6.3	7	180.9±9.26	7	149.5±6.09	6	156.2±10.06	6	152.6±7.36	7	185.5±12.96	6
12	147.6±5.47	7	191.5±14.75 **	7	145.9±7.18	6	164.3±10.93	6	154±7.39	7	191±14.34	6
13	148.8±5.56	7	181.8±11.85	6	148.2±7.57	6	163.6±12.11	6	156.4±7.52	7	191±14.48	6
14	156.9±8.05	7	183.7±11.25	6	148.4±7.71	6	163.1±11.2	6	162.4±9.16	7	194.4±15.1	6
15	158.8±6.79	7	198.3±12.67 **	6	146.4±6.82	6	172±14.05	6	164.3±6.9	7	201.9±14.76	6
16	163.4±6.51	7	208.3±13.25 **	6	152.8±9.87	6	177.4±13.39	6	164.8±9.45	7	208.8±16.71 *	6
17	160.4±5.36	7	206.2±14.95 **	6	152.9±8.29	6	178.7±12.21	6	169.9±6.69	7	215.4±10.6 *	6
18	163.6±6.1	7	208.6±15.52 **	6	155.2±9.28	6	186.7±19.11	6	169.4±6.76	7	208.3±14.33 *	6
19	161±6.15	7	215.5±14.96 ***	6	160.4±5.22	6	184.2±14.06	6	174.1±6.84	7	221.1±14.05 *	6
20	163.8±9.32	7	216.5±13.68 ***	6	154.7±7.11	6	186.5±16.16	6	170.1±9.01	7	220.3±16.58 **	6
21	161.3±7.2	7	219.7±12.16 ***	6	161.1±7.28	4	181.5±10.91	6	170.5±6.71	7	225.7±16.31 **	6
22	166.1±10.45	7	224±12.22 ***	6	156.4±6.41	6	188±15.82	6	174.4±7.76	7	234±15.99 ***	6
23	164.1±8.62	7	230.2±10.98 ***	6	161.6±5.43	6	194.5±15.67	6	175.4±8.4	7	235±18.57 ***	6
24	162.5±8.25	7	236.5±11.7 ***	6	159.1±6.95	6	199.6±20.11 *	6	179.2±9.18	7	232.6±19.14 ***	6
25	166.3±9.49	7	237.6±15.46 ***	6	154.2±5.97	6	199.2±19.05 *	6	177.5±8.46	7	238.7±17.23 ***	6
26	169.7±7.55	7	242.3±14.82 ***	6	158±4.87	6	200.8±20.58 *	6	179.1±7.27	7	233.6±19.27 ***	6

LFD, low-fat diet. HFD, high-fat diet. Statistics: two-way AOVA of repeated measurements assessing the overall effect of diet, time, and their interaction, followed by Bonferroni post-test to assess diet effect: * p<0.05, ** p<0.01, *** p<0.001.

Supplemental Table S13: Gained body mass at study endpoint (26 weeks) in male and female WT, OI, and OI/OCN-KO mice.

Statistics	Male mice<br>genotype: 0.06. diet: <0.001. interaction: 0.001				Female mice<br>genotype: 0.01. diet: <0.001. interaction: n.s.			
**Diet**	**LFD**		**HFD**		**LFD**		**HFD**	
	Mean±SEM	N	Mean±SEM	N	Mean±SEM	N	Mean±SEM	N
**WT**	74.6±11.53	8	159.5±9.33	6	76.7±9.47	7	155±17.87	7
**OI**	87.3±9.6	6	108.5±9.58 §	6	56.3±8.77	8	132.7±17.9	6
**OI/OCN-KO**	67.9±8.48	7	188.9±20.87 ###	6	64.8±7.81	7	150.8±16.17	7

LFD, low-fat diet. HFD, high-fat diet. Statistics: Within each sex, two-way ANOVA was applied to assess the overall effect of genotype, diet, and their interaction, followed by Bonferroni post-test within each diet to assess genotype effect by comparison to diet-matched WT: § p<0.05, and to diet-matched OI: ### p<0.001.

Supplemental Table S14: Adipose tissue volume over tissue volume (AV/TV) at dietary study endpoint (26 weeks) in male and female WT, OI, and OI/OCN-KO mice.

Statistics	Male mice<br>genotype: n.s. diet: <0.001. interaction: n.s.				Female mice<br>genotype: 0.01. diet: <0.001. interaction: n.s.			
**Diet**	**LFD**		**HFD**		**LFD**		**HFD**	
	Mean±SEM	N	Mean±SEM	N	Mean±SEM	N	Mean±SEM	N
**WT**	17±2.85	8	41.1±2.97 ***	6	21.2±5.39	7	56±3.87 ***	6
**OI**	12.5±3.34	5	27.6±6.13	6	13.3±1.82	8	31.6±8.83 §	6
**OI/OCN-KO**	12.9±1.64	7	37.2±4.72 ***	6	11.9±1.18	6	44.2±7.03 **	7

LFD, low-fat diet. HFD, high-fat diet. Statistics: Within each sex, two-way ANOVA assessing the overall effect of genotype, diet, and their interaction, followed by Bonferroni post-test to assess diet effects: ** p<0.01, *** p<0.001, genotype effect by comparison to diet-matched WT: § p<0.05, and to diet-matched OI: n.s..

Supplemental Table S15: Glucose tolerance testing (GTT) in 4-week intervals during a 26-week dietary intervention in male and female WT, OI, and OI/OCN-KO mice. Absolute values are shown. Statistics are indicated in the respective figure.

**Genotype**	**WT**								**OI**								**OI/OCN-KO**							
**Diet**	**LFD**				**HFD**				**LFD**				**HFD**				**LFD**				**HFD**			
Male mice																								
**Diet (weeks)**	**Median**	**P90**	**P10**	**N**	**Median**	**P90**	**P10**	**N**	**Median**	**P90**	**P10**	**N**	**Median**	**P90**	**P10**	**N**	**Median**	**P90**	**P10**	**N**	**Median**	**P90**	**P10**	**N**
0	3.14	3.19	3.09	10	3.14	3.19	3.09	10	3.12	3.19	3	10	3.12	3.19	3	10	3.1	3.22	3.02	9	3.1	3.22	3.02	9
4	3.2	3.25	3.16	7	3.28	3.37	3.22	4	3.21	3.27	3.05	7	3.21	3.33	3.17	5	3.22	3.27	3.1	8	3.3	3.34	3.09	5
8	3.27	3.34	3.13	7	3.37	3.39	3.25	6	3.12	3.3	3	7	3.2	3.43	3.13	6	3.18	3.38	3.01	9	3.31	3.45	3.17	5
12	3.2	3.3	3.15	8	3.36	3.52	3.14	6	3.1	3.27	3.03	5	3.2	3.42	3.16	5	3.19	3.34	3.04	8	3.31	3.54	3.22	5
16	3.18	3.33	3.09	8	3.37	3.58	3.32	6	3.13	3.24	3.01	6	3.25	3.44	3.12	6	3.19	3.23	3.06	7	3.34	3.53	3.25	5
20	3.19	3.49	3.11	8	3.33	3.5	3.24	5	3.11	3.31	3.02	5	3.25	3.37	2.92	6	3.16	3.29	3.02	6	3.36	3.45	3.23	5
24	3.19	3.5	3.11	8	3.37	3.48	3.26	6	3.14	3.29	3	6	3.3	3.53	3.04	6	3.11	3.31	3.05	7	3.35	3.55	3.2	5
Female mice																								
**Diet (weeks)**	**Median**	**P90**	**P10**	**N**	**Median**	**P90**	**P10**	**N**	**Median**	**P90**	**P10**	**N**	**Median**	**P90**	**P10**	**N**	**Median**	**P90**	**P10**	**N**	**Median**	**P90**	**P10**	**N**
0	3.09	3.14	3.05	7	3.09	3.14	3.05	6	3.02	3.23	2.87	10	3.02	3.23	2.87	10	3.09	3.23	2.89	7	3.09	3.23	2.89	7
4	3.13	3.27	3.02	7	3.16	3.33	3.06	6	3.11	3.22	2.97	6	3.2	3.32	3.05	6	3.08	3.23	2.88	7	3.18	3.31	3	6
8	3.05	3.25	3.02	7	3.17	3.27	3.11	6	3.08	3.27	2.91	6	3.23	3.28	3.22	6	3.04	3.25	2.86	7	3.23	3.32	3.12	6
12	3.08	3.28	2.92	7	3.21	3.34	3.1	6	3.12	3.3	2.93	6	3.21	3.35	3.16	6	3.06	3.14	2.95	7	3.2	3.32	3.06	6
16	3.18	3.34	3.12	7	3.23	3.34	3.17	6	3.06	3.41	2.98	6	3.2	3.39	3.17	6	3.03	3.21	2.99	7	3.27	3.35	3.01	6
20	3.1	3.32	3.04	7	3.26	3.37	3.14	6	3.1	3.25	2.99	6	3.21	3.34	3.04	6	3.07	3.22	2.98	7	3.21	3.33	3.1	6
24	3.08	3.26	2.99	7	3.22	3.38	3.16	6	3.03	3.16	3	6	3.18	3.3	2.98	6	3.04	3.22	2.98	7	3.21	3.37	3.07	6

LFD, low-fat diet. HFD, high-fat diet. The GTT area under the curve (AUC) was calculated using the trapezoid rule [58] for each time point and then log10-transformed. Transformed data are presented with median, 90th and 10th percentile (P90/P10) and N per diet time point, sex, and genotype.

Supplemental Table S16: Insulin tolerance testing (ITT) at dietary study endpoint in male and female WT, OI, and OI/OCN-KO mice. Absolute values are shown. Statistics are indicated in the respective figure.

**Genotype**		**WT**				**OI**				**OI/OCN-KO**			
**Diet**		**LFD**		**HFD**		**LFD**		**HFD**		**LFD**		**HFD**	
Male mice													
		**Mean±SEM**	**N**	**Mean±SEM**	**N**	**Mean±SEM**	**N**	**Mean±SEM**	**N**	**Mean±SEM**	**N**	**Mean±SEM**	**N**
**Statistics**		diet: <0.001. time: <0.001. interaction: n.s.				diet: n.s. time: n.s. interaction: n.s.				diet: 0.001. time: 0.03. interaction: 0.05.			
**Time (min)**	**0**	100±0	6	100±0	5	100±0	5	100±0	4	100±0	6	100±0	3
	**15**	77.3±8.9	8	98.5±7.2	5	121.1±17.3	5	104.9±12.5	4	104.4±12.1	5	62.9±11.2 *	4
	**30**	66.7±10.2	8	60.6±5.8	5	112.2±17.7	5	91.9±13.9	4	93.1±13.1	6	42.5±3.7 **	4
	**60**	66.2±10.1	8	63.3±5.3	5	111.2±8.5	5	113.2±17.4	4	88.5±10.7	6	52.9±4.2	4
	**90**	75.3±15.4	8	82.3±8.1	5	97.6±16.7	5	112.7±15.5	4	97.6±11.6	6	60.1±7.3	4
	**120**	81.6±11.4	8	88.6±8.1	5	94±15.6	5	110.8±10.3	4	103.8±13.8	6	70.2±9.9	4
Female mice													
		**Mean±SEM**	**N**	**Mean±SEM**	**N**	**Mean±SEM**	**N**	**Mean±SEM**	**N**	**Mean±SEM**	**N**	**Mean±SEM**	**N**
**Statistics**		diet: n.s. time: <0.001. interaction: n.s.				diet: n.s. time: 0.002. interaction: n.s.				diet: n.s. time: n.s. interaction: n.s.			
**Time (min)**	**0**	100±0	5	100±0	5	100±0	6	100±0	4	100±0	5	100±0	7
	**15**	78±4.8	5	79.6±10.1	5	83.2±10.5	6	88.8±11.1	4	81.2±15	6	96.5±9.5	7
	**30**	69.1±4.7	5	69±6.8	5	71.2±11.9	6	66.6±7.7	4	71.6±9.4	6	75.5±10.7	7
	**60**	82.9±11.4	5	75.3±11.6	5	62.4±15.3	6	66.5±6.9	4	71.9±12.9	6	86.9±6.9	7
	**90**	97.1±13.9	5	74.6±9.1	5	70.7±14.8	6	71.4±6.6	4	80.8±15.5	6	95.3±5.7	7
	**120**	97.4±7.5	5	88.6±5.6	5	76.1±13.1	6	76.3±3.9	4	82.2±17.2	6	98.5±6.8	7

LFD, low-fat diet. HFD, high-fat diet. ITT measurements are shown as changes in glucose levels (% from initial glucose levels) after insulin injection at time point 0. Statistics: two-way ANOVA of repeated measures assessing the overall effect of diet, time, and their interaction, followed by Bonferroni post-test to assess diet effect per time point: * p<0.05, ** p<0.01

Supplemental Table S17: ITT (AUC) at dietary study endpoint in male and female WT, OI, and OI/OCN-KO mice.

**Sex**	**Male mice**				**Female mice**			
**Statistics**	genotype: 0.01. diet: n.s. interaction: n.s.				genotype: n.s. diet: n.s. interaction: n.s.			
**Diet**	**LFD**		**HFD**		**LFD**		**HFD**	
**Genotype**	Mean±SEM	N	Mean±SEM	N	Mean±SEM	N	Mean±SEM	N
**WT**	9512±1124	8	9292±720	6	10343±969.7	5	9327±835.5	5
**OI**	12769±1326	5	12718±1132	5	8738±1468	6	8867±701.9	4
**OI/OCN-KO**	11661±1298	6	7097±520.6 #	4	9401±1320	6	10846±713.4	7

LFD, low-fat diet. HFD, high-fat diet. The area under the curve (AUC) was calculated using the trapezoid rule [58] based on the measurements shown in Supplemental Table S15. Statistics: Within each sex, two-way ANOVA assessing the overall effect of genotype, diet, and their interaction, followed by Bonferroni post-test to assess diet effects: n.s., genotype effect by comparison to diet-matched WT: n.s., and to diet-matched OI: # p<0.05.

Supplemental Table S18: Pancreatic insulin levels assessed after 26 weeks of low-fat (LFD) or high-fat diet (HFD).

	Absolute pancreatic insulin levels (mM)				Relative pancreatic insulin levels (mM/g tissue)			
**Diet**	**LFD**		**HFD**		**LFD**		**HFD**	
	Mean±SEM	N	Mean±SEM	N	Mean±SEM	N	Mean±SEM	N
Male mice								
**Statistics**	genotype: 0.02. diet: n.s. interaction: n.s.				genotype: n.s. diet: 0.08 interaction: 0.02			
**WT**	2.1±0.42	6	2.2±0.36	5	4.7±1.00	6	2.1±0.29 *	5
**OI**	1.4±0.25	5	1.6±0.24	6	3.4±0.62	5	3.4±0.38	6
**OI/OCN-KO**	1.0±0.08 *(vs WT p=0.07)*	7	1.7±0.31	5	2.8±0.18	7	3.1±0.60	5
Female mice								
**Statistics**	genotype: n.s. diet: n.s. interaction: n.s.				genotype: n.s. diet: n.s. interaction: 0.006			
**WT**	1.7±0.24	7	1.4±0.34	6	4.6±0.76	7	1.7±0.49 *	6
**OI**	1.5±0.29	5	1.7±0.39	7	3.4±0.56	5	5.0±0.71 §	7
**OI/OCN-KO**	1.5±0.23	7	1.7±0.49	7	3.7±0.677	7	3.8±0.43	7

Statistical analysis: For each outcome measure: two-way ANOVA assessing the overall effect of genotype, diet, and their interaction, followed by Bonferroni post-test to assess diet effects: * $p<0.05$, genotype effect by comparison to diet-matched WT: § $p<0.05$, and to diet-matched OI: n.s.

Supplemental Table S19: Indirect calorimetry assessment of male and female WT, OI, and OI/OCN-KO mice after 22 weeks of dietary intervention. Absolute values are shown. Statistics are indicated in the respective figure.

	**WT**				**OI**				**OI/OCN-KO**			
	**LFD**		**HFD**		**LFD**		**HFD**		**LFD**		**HFD**	
Male mice												
	**LOMO**	**HIMO**	**LOMO**	**HIMO**	**LOMO**	**HIMO**	**LOMO**	**HIMO**	**LOMO**	**HIMO**	**LOMO**	**HIMO**
**VCO_2 (ml/kg/hr)**	3155±107.6; 5	4122±187.8; 6	2076±371.6; 4	2390±211.5; 4	4043±51.46; 3	4270±392.9; 4	3186±250.4; 4	3740±39.5; 3	3629±313.6; 5	4469±246.5; 5	2734±191; 5	2731±196.4; 5
**VO_2 (ml/kg/hr)**	3413±82.48; 5	4585±272.5; 6	2830±292.7; 4	3240±197.8; 4	4403±132; 4	4673±320; 4	4124±343.5; 4	4924±56.55; 3	4355±187.2; 5	4783±132.9; 5	3559±202.9; 5	3587±245.5; 5
**RER (VCO_2/VO_2)**	0.91±0.019; 6	0.91±0.03; 6	0.79±0.02; 3	0.74±0.05; 4	0.87±0.03; 4	0.91±0.03; 4	0.77±0.004; 4	0.77±0.01; 4	0.83±0.05; 5	0.93±0.04; 5	0.76±0.01; 5	0.76±0.003; 5
**Energy expenditure (kcal/hr/kg)**	17.6±0.39; 5	22.4±1.26; 6	12.9±1.66; 4	15.4±0.95; 4	21.7±0.41; 4	23.4±1.76; 4	19.3±1.67; 4	23.1±0.11; 3	21±1.05; 5	23.4±0.73; 5	16.8±0.98; 5	16.9±1.21; 5
**Food (g)**	0.13±0.02; 6	0.23±0.11; 5	0.01±0.005; 4	0.02±0.01; 4	0.06±0.03; 4	0.10±0.02; 4	0.06±0.03; 4	0.03±0.02; 4	0.06±0.03; 4	0.09±0.04; 5	0.09±0.03; 5	0.05±0.02; 5
Female mice												
	**LOMO**	**HIMO**	**LOMO**	**HIMO**	**LOMO**	**HIMO**	**LOMO**	**HIMO**	**LOMO**	**HIMO**	**LOMO**	**HIMO**
**VCO_2 (ml/kg/hr)**	3755±435.1; 5	4350±477.8; 5	2853±481; 4	3580±562.6; 4	4148±426.8; 5	4899±270.8; 5	3078±300.6; 4	3969±461.8; 4	4295±271.2; 6	5085±377.1; 6	3326±211.9; 5	3737±256.2; 5
**VO_2 (ml/kg/hr)**	3980±214.1; 5	4956±267.4; 5	3668±535.1; 4	4816±527.9; 4	4921±448.3; 5	5378±302.2; 5	4820±621.1; 4	5405±674; 4	5069±291.1; 6	5977±454.6; 6	4253±265; 5	4552±274.6; 5
**RER (VCO_2/VO_2)**	0.84±0.07; 5	0.92±0.02; 4	0.78±0.007; 3	0.78±0.01; 3	0.84±0.03; 5	0.91±0.02; 5	0.76±0.01; 4	0.77±0.01; 4	0.85±0.05; 6	0.87±0.05; 6	0.76±0.02; 5	0.77±0.01; 5
**Energy expenditure (kcal/hr/kg)**	19.6±1.30; 5	24.0±1.54; 5	17.2±2.65; 4	23.4±2.86; 4	23.4±2.06; 5	26.5±1.17; 5	22.8±2.72; 4	25.9±3.39; 4	24.5±1.25; 6	28.9±1.94; 6	20.4±1.19; 5	21.8±1.32; 5
**Food (g)**	0.04±0.01; 5	0.09±0.02; 4	0.05±0.02; 4	0.1±0.05; 4	0.08±0.01; 4	0.09±0.04; 5	0.05±0.02; 4	0.13±0.06; 4	0.09±0.02; 5	0.15±0.03; 6	0.018±0.01; 5	0.04±0.02; 5

LFD, low-fat diet. HFD, high-fat diet. Data are shown as mean±SEM;N. Mice were housed in the indirect calorimetry chambers for 4 consecutive days. Days 1 to 3 were used as the acclimatization phase for the mice to the chambers and day 4 for final analysis. For analysis, metabolic, feeding, and locomotion measurements behavior were averaged for 4 day-time periods (06:00 am - 08:59 am, period 1; 9:00 am - 11:59 am, period 2; 12 pm - 2:59 pm, period 3; 03:00 pm - 05:59 pm, period 4) and for 4 night-time periods (06:00 pm - 08:59 pm, period 5; 09:00 pm - 11:59 pm, period 6; 12:00 am - 02:59 am, period 7; 03:00 - 05:59 am, period 8) of day 4. As observed before, mice with OI genotype exhibit significantly altered locomotion compared to their wild-type counterparts, likely due to spontaneous fractures and pain [16,59]. To minimize variations within measurements caused by differences in locomotion activity, we identified periods with activity levels of low mobility (LOMO) and high mobility (HIMO) for each genotype and dietary intervention and used these periods for further analysis of the metabolic phenotype.

Supplemental Table S20: Body surface temperature (ºC) in male WT, OI, and OI/OCN-KO mice during dietary intervention.
Absolute values are shown. Statistics are indicated in the respective figure.

Genotype	WT				OI				OI/OCN-KO			
Diet	LFD		HFD		LFD		HFD		LFD		HFD	
Weeks of diet	Mean±SEM	N	Mean±SEM	N	Mean±SEM	N	Mean±SEM	N	Mean±SEM	N	Mean±SEM	N
0	35.2±0.1	4	35.2±0.14	3	35.2±0.04	7	35.4±0.12	4	35.2±0.07	7	35.2±0.07	4
1	35.1±0.03	8	35.2±0.07	4	35.2±0.03	5	35.3±0.09	5	35.1±0.05	8	35.3±0.06	4
2	35.1±0.04	6	35.3±0.07	6	35.2±0.04	6	35.2±0.07	5	35.1±0.04	8	35.1±0.03	3
3	35.2±0.05	8	35.2±0.07	5	35.2±0.06	5	35.4±0.04	6	35.2±0.07	9	35.1±0.05	5
4	35.1±0.04	7	35.2±0.03	6	35.2±0.05	5	35.3±0.08	5	35.2±0.04	6	35.1±0.08	4
5	35.1±0.04	7	35.1±0.06	3	35.1±0.05	5	35.2±0.12	4	35.1±0.06	7	35.1±0.12	3
6	35.3±0.07	5	35.2±0.07	4	35.1±0.06	4	35.3±0.04	5	35.1±0.05	7	35±0	3
7	35.1±0.04	5	35.1±0.04	4	35.1±0.08	4	35.1±0.03	3	35.1±0.09	6	35.1±0.05	2
8	35.3±0.07	6	35.1±0.04	4	35.1±0.14	2	35.2±0.07	4	35.2±0.03	7	35.2±0	1
9	35.2±0.05	7	35.2±0.03	3	35.1±0.09	5	35.2±0.07	4	35.1±0.04	6	35.1±0.03	3
10	35.2±0.1	5	35.2±0.04	2	35.1±0.08	3	35.3±0.07	6	35.1±0.02	7	35.2±0.09	3
11	35.2±0.06	6	35.2±0.07	5	35.2±0.1	4	35.3±0.08	5	35.1±0.04	6	35.1±0.05	2
12	35.2±0.05	5	35.1±0.04	5	35.1±0.06	5	35.2±0.05	5	35.2±0.06	5	35.2±0.05	3
13	35.1±0.04	8	35.2±0.07	5	35±0.02	5	35.1±0.06	4	35.1±0.04	4	35.1±0.08	4
14	35.1±0.05	8	35.1±0.04	4	35.1±0.05	3	35.1±0.02	4	35.1±0.08	3	35.1±0.06	4
15	35.2±0.03	6	35.1±0.03	5	35.1±0	2	35.2±0.04	4	35.1±0	2	35.2±0.09	2
16	35.1±0.07	7	35.1±0	3	35.1±0.04	4	35.1±0.02	5	35±0.05	2	35.2±0	1
17	35.2±0.08	8	35.1±0.08	4	35.1±0.04	4	35.2±0.05	3	35±0.02	4	35.1±0.08	3
18	35.3±0.07	7	35.1±0.07	5	35.1±0.04	4	35.1±0.06	4	35±0.02	4	35±0.03	3
19	35.2±0.06	6	35.1±0.07	5	35.1±0	2	35±0.05	2	35.1±0.03	5	NM	
20	35.2±0.04	4	35.1±0.08	3	35.3±0.09	2	35.2±0.15	3	35.1±0.06	3	35±0.03	3
21	35.4±0.08	3	35.1±0.06	4	35±0.05	2	35.1±0.05	3	35.1±0.04	4	NM	
22	35.1±0.06	3	35.1±0.08	4	35.1±0.14	2	35.4±0	1	35.1±0.02	4	35.1±0.05	2
23	35.1±0.13	3	35.1±0.06	3	35±0.05	2	35.2±0.09	4	35.1±0.03	5	35±0	1
24	35.3±0.04	4	NM		35±0	1	35±0.03	3	35.1±0	2	35.1±0.1	2
25	NM		35.2±0.08	4	35.2±0	1	35±0.05	2	35.1±0	2	35.1±0	1
26	35.2±0.08	3	35.1±0.05	5	35.1±0	2	35±0.02	4	35.2±0.07	5	35.2±0.09	2

LFD, low-fat diet. HFD, high-fat diet. NM, not measured. Body surface temperature was measured at the anterior side under the diaphragm weekly between 15-16 o'clock.

Supplemental Table S21: Body surface temperature (ºC) in female WT, OI, and OI/OCN-KO mice during dietary intervention. Absolute values are shown. Statistics are indicated in the respective figure.

Genotype	WT				OI				OI/OCN-KO			
Diet	LFD		HFD		LFD		HFD		LFD		HFD	
**Weeks of diet**	**Mean±SEM**	**N**	**Mean±SEM**	**N**	**Mean±SEM**	**N**	**Mean±SEM**	**N**	**Mean±SEM**	**N**	**Mean±SEM**	**N**
**0**	35.4±0.06	6	35.3±0.05	8	35.2±0.11	5	35.5±0.05	4	35.4±0.08	8	35.5±0.09	4
**1**	35.2±0.06	7	35.3±0.07	7	35±0.02	7	35.4±0.07	6	35.3±0.04	8	35.4±0.05	6
**2**	35.2±0.06	6	35.4±0.06	9	35.1±0.04	8	35.5±0.06	6	35.2±0.05	8	35.3±0.05	7
**3**	35.3±0.05	6	35.3±0.08	9	35.1±0.03	7	35.4±0.11	6	35.1±0.03	8	35.4±0.13	6
**4**	35.5±0.08	5	35.2±0.07	8	35.2±0.09	6	35.3±0.1	5	35.3±0.05	8	35.5±0.08	6
**5**	35.3±0.05	7	35.3±0.05	7	35.2±0.04	6	35.4±0.05	5	35.2±0.06	8	35.5±0.12	7
**6**	35.3±0.08	7	35.3±0.05	9	35.2±0.05	6	35.3±0.08	6	35.1±0.09	6	35.4±0.09	6
**7**	35.3±0.09	7	35.3±0.07	8	35.2±0.03	6	35.4±0.08	6	35.3±0.04	8	35.4±0.08	5
**8**	35.2±0.08	6	35.2±0.08	8	35.2±0.08	6	35.3±0.03	6	35.2±0.07	6	35.1±0.09	5
**9**	35.4±0.08	5	35.4±0.09	7	35.2±0.06	7	35.3±0.14	6	35.2±0.04	8	35.2±0.1	5
**10**	35.2±0.05	5	35.2±0.07	7	35.2±0.07	8	35.2±0.09	5	35.2±0.07	6	35.2±0.07	4
**11**	35.2±0.07	5	35.3±0.1	7	35.2±0.09	6	35.4±0.09	6	35.1±0.02	8	35.2±0.07	6
**12**	35.4±0.07	5	35.2±0.04	7	35.2±0.14	6	35.3±0.07	6	35.2±0.1	7	35.3±0.06	5
**13**	35.3±0.1	5	35.3±0.06	7	35.2±0.09	6	35.2±0.07	6	35.2±0.1	6	35.1±0.08	4
**14**	35.2±0.06	6	35.3±0.04	7	35.2±0.04	8	35.3±0.07	6	35.2±0.05	5	35.2±0.08	6
**15**	35.3±0.1	6	35.3±0.13	5	35.1±0.04	7	35.3±0.04	6	35.2±0.1	7	35.2±0.07	4
**16**	35.4±0.1	3	35.3±0.07	6	35.1±0.06	4	35.2±0.05	3	35.3±0.17	5	35.1±0.1	2
**17**	35.3±0.09	5	35.3±0.07	5	35.3±0.09	6	35.1±0.06	5	35.3±0.05	6	35.3±0.17	3
**18**	35.1±0.07	5	35.1±0.11	4	35.5±0.1	3	35.1±0.03	5	35.3±0.11	7	35.2±0.12	5
**19**	35.2±0.08	5	35.3±0.1	6	35.4±0.12	3	35.1±0.07	5	35.3±0.03	5	35.2±0.13	6
**20**	35.3±0.09	5	35.2±0.09	5	35.2±0.09	6	35.2±0.14	3	35.4±0.09	4	NM	
**21**	35.6±0.18	4	35.3±0.08	5	35.4±0.2	3	35.2±0.09	5	35.4±0.2	4	35.4±0.15	3
**22**	35.4±0.12	3	35.3±0.07	5	35.2±0.08	4	35.2±0.05	3	35.3±0.09	6	NM	
**23**	35.1±0.05	5	35.1±0.07	4	35.1±0.06	4	35.4±0.09	4	35.2±0.08	6	35.3±0.06	4
**24**	35.2±0.08	3	35.2±0.06	4	35.1±0.14	2	35.2±0.08	4	35.2±0.1	4	35.2±0.07	5
**25**	35.3±0	2	NM		35.2±0	1	35±0	1	35.1±0.05	2	35±0.06	3
**26**	NM		35.3±0.05	2	35.3±0.12	6	35.2±0	4	35.2±0.1	5	35.2±0.07	5

LFD, low-fat diet. HFD, high-fat diet. NM, not measured. Body surface temperature was measured at the anterior side under the diaphragm weekly between 15-16 o'clock.

Supplemental Table S22: Absolute organ masses (g) after 26 weeks of low-fat/high-fat diet.

		WT		OI		OI/OCN-KO	
**Organ**	**Statistics**	**LFD**	**HFD**	**LFD**	**HFD**	**LFD**	**HFD**
Male mice		N=8	N=6	N=6	N=6	N=7	N=6
**Liver**	genotype: 0.002<br>diet: n.s.<br>interaction: n.s.	1.75±0.19	1.80±0.08	1.36±0.09	1.33±0.07	1.25±0.02	1.41±0.15
**Gastrocnemius**	genotype: <0.001<br>diet: n.s.<br>interaction: n.s.	0.23±0.02	0.21±0.01	0.13±0.01 §§§	0.15±0.01 *(vs WT HFD: 0.07)*	0.14±0.01 §§	0.13±0.01 §
**Pancreas**	genotype: 0.007<br>diet: <0.001<br>interaction: 0.03	0.45±0.06	1.00±0.07 ***	0.39±0.04	0.49±0.07 §§	0.37±0.03	0.74±0.16 *(vs LFD: p=0.06)*
**Brown adipose tissue**	genotype: 0.007<br>diet: 0.001<br>interaction: n.s.	0.18±0.02	0.34±0.06 *	0.11±0.01	0.17±0.03 §	0.14±0.01	0.23±0.06
**White adipose tissue**	genotype: 0.02<br>diet: <0.001<br>interaction: n.s.	0.47±0.05	1.70±0.26 ***	0.30±0.06	0.69±0.13 §§	0.35±0.04	1.24±0.36 *
Female mice		N=7	N=7	N=8	N=5	N=7	N=7
**Liver**	genotype: 0.06<br>diet: n.s.<br>interaction: n.s.	1.22±0.06	1.47±0.14	1.23±0.09	1.15±0.10	1.04±0.10	1.16±0.08
**Gastrocnemius**	genotype: 0.08<br>diet: n.s.<br>interaction: n.s.	0.16±0.002	0.18±0.01	0.13±0.02	0.13±0.004	0.13±0.01	0.17±0.02
**Pancreas**	genotype: n.s.<br>diet: 0.03<br>interaction: 0.08	0.39±0.04	0.70±0.17	0.41±0.05	0.34±0.05	0.34±0.02	0.62±0.10
**Brown adipose tissue**	genotype: 0.01<br>diet: 0.01<br>interaction: 0.06	0.11±0.01	0.22±0.05 *	0.09±0.01	0.07±0.01 §§	0.09±0.01	0.16±0.03
**White adipose tissue**	gentotype: n.s.<br>diet: <0.001<br>interaction: n.s.	0.50±0.05	1.66±0.39*	0.37±0.04	1.07±0.45	0.31±0.03	1.28±0.32

Data are means±SEM. LFD, low-fat diet. HFD, high-fat diet. Interscapular brown adipose tissue and inguinal white adipose tissue were analysed. Statistical analysis: two-way ANOVA within each sex to assess overall effect of genotype, the overall diet effect, and their interaction, followed by Bonferroni post-test to assess diet effect: * p<0.05. ** p< 0.01. *** p<0.001; genotype effect compared to diet-matched WT: § p<0.05, or genotype compared to diet-matched OI: n.s.

.

Supplemental Table S23: Overview of total movement of mice fed either regular chow or custom diets. Time points selected for subsequent metabolic phenotype analysis are marked in bold-italic and cell shading with yellow indicating low mobility, LOMO or blue indicating high mobility, HIMO.

	**Sex**	**Male mice on regular chow**					
	**Genotype**	**WT**		**OI**		**OI/OCN-KO**	
	**Age**	**Juvenile (N=6)**	**Adolescent (N=11)**	**Juvenile (N=6)**	**Adolescent (N=8)**	**Juvenile (N=9)**	**Adolescent (N=15)**
	**Time period**	Mean±SEM	Mean±SEM	Mean±SEM	Mean±SEM	Mean±SEM	Mean±SEM
**Day**	**06:00-08:59 am**	3785,5±785,6	2059,7±350	3651±775,2	1751,2±191,3	2098,4±375,7	2208,3±417,3
	**09:00-11:59 am**	3384,5±761,8	1912,1±338,9	***3828,2±985***	1677,8±248,1	2794,9±526,8	***2014,9±398,8***
	**12:00-02:59 pm**	2947,3±660,8	2041±340,6	3759,6±626,1	1527,8±219,2	2064,4±351,7	2142,3±411,9
	**03:00-05:59 pm**	***1961,5±384***	***1609,6±335,5***	3031,4±406,3	***1490±143***	***2009,1±441,7***	2428,8±422,8
**Night**	**06:00-08:59 pm**	***4375,4±580,2***	2459,3±317,7	3246,9±363	***2671,4±480,8***	2873,3±318,9	***3240,2±447,7***
	**09:00-11:59 pm**	3958,8±294,2	***2740,7±261,2***	3779,1±551,6	2525,7±259,3	3033,2±225,2	3002,4±350,2
	**12:00-02:59 am**	2837,6±439,6	2108,4±314,1	***2798,7±327,2***	2534±240,3	***3617,5±494,7***	2265,6±287,6
	**03:00-05:50 am**	3362,6±582,4	1921,6±182,5	3813,1±881	2102,1±257,8	3097,1±478,3	2355,8±316,7
	**Sex**	**Female mice on regular chow**					
	**Genotype**	**WT**		**OI**		**OI/OCN-KO**	
	**Age**	**Juvenile (N=8)**	**Adolescent (N=7)**	**Juvenile (N=8)**	**Adolescent (N=12)**	**Juvenile (N=9)**	**Adolescent (N=13)**
	**Time period**	Mean±SEM	Mean±SEM	Mean±SEM	Mean±SEM	Mean±SEM	Mean±SEM
**Day**	**06:00-08:59 am**	2064,4±1784,7	1758±686,2	3137,6±1896,5	2892,3±1362,5	3574,5±2130	***2264,1±836,2***
	**09:00-11:59 am**	2217±1398,3	***1585±659,9***	3224,8±1663,3	2214,6±1194,5	3425,6±1857,3	2279,1±739,1
	**12:00-02:59 pm**	2058,8±1416,5	1935,4±1100,2	***2205,9±892,1***	2505,1±1065,1	3899,1±2511	2567±1237,3
	**03:00-05:59 pm**	2068,2±1411	1731,8±1272,6	2360,9±1455,9	***2134,1±827,7***	***3331,7±1527,7***	2437,1±1212,6
**Night**	**06:00-08:59 pm**	2339,4±1668,7	2289,1±941,2	2962,9±889,8	3072,1±1037,6	3804,7±1358,1	3333±1101,9
	**09:00-11:59 pm**	***2883,7±1760,7***	***3565±1077,5***	3372,1±1564	***3843,8±1181,2***	3927,1±989,9	***3873±1180,8***
	**12:00-02:59 am**	2422±1600,8	2052,5±1094,8	***3503±849,7***	2841,3±1204,5	4224,5±1916,3	3020,5±1367,8
	**03:00-05:50 am**	***1807,2±1368,3***	1815,1±807,2	3295,7±1172,3	2972,3±1489,5	***4446,4±2082,6***	2475,1±914,1
	**Sex**	**Male mice on custom diets**					
	**Genotype**	**WT**		**OI**		**OI/OCN-KO**	
	**Diet**	**LFD (N=6)**	**HFD (N=4)**	**LFD (N=4)**	**HFD (N=4)**	**LFD (N=5)**	**HFD (N=5)**
	**Time period**	Mean±SEM	Mean±SEM	Mean±SEM	Mean±SEM	Mean±SEM	Mean±SEM

**Day**	**06:00-08:59 am**	1730,4±381,8	1966,9±1265,6	1099,5±415,5	1306,9±331,9	2498,1±445,7	1977,1±654,4
	**09:00-11:59 am**	1683,2±225,6	***1066,4±579,9***	1161,2±329,6	1095±317	1638,3±225,7	***2392,4±892,5***
	**12:00-02:59 pm**	2001,2±427,1	2176,4±1045	***1002,6±214***	1079,5±243,6	***1450,7±350,9***	2346,8±842
	**03:00-05:59 pm**	***1517,3±258,8***	***2505,2±1119***	1116,9±141,9	***1063,4±270,8***	1863,2±214,4	***1565,3±511***
**Night**	**06:00-08:59 pm**	2247,6±242,4	2203,6±883,7	1233,5±363,5	1144,9±387,8	***2513,3±459,7***	2360,7±486,7
	**09:00-11:59 pm**	***2584±468,1***	2215,4±955,9	1177±290,3	1624,7±425,2	2403,5±304,9	2387,5±276
	**12:00-02:59 am**	2058,9±273,5	2269±1094,7	1092±389,1	***1872,7±520,1***	2241,1±344	1574,9±379,2
	**03:00-05:50 am**	1634,1±287,7	1959,9±1007	***1281,8±392,2***	1355±288,4	2264,8±472,4	2268,6±716,9

	**Sex**	**Female mice on custom diets**					
	**Genotype**	**WT**		**OI**		**OI/OCN-KO**	
	**Diet**	**LFD (N=5)**	**HFD (N=4)**	**LFD (N=5)**	**HFD (N=4)**	**LFD (N=6)**	**HFD (N=5)**
	**Time period**	Mean±SEM	Mean±SEM	Mean±SEM	Mean±SEM	Mean±SEM	Mean±SEM
**Day**	**06:00-08:59 am**	2669,1±558,6	1836,9±259,8	2662,8±586,6	1077,7±392,6	2612,2±418,4	1437,5±213,5
	**09:00-11:59 am**	***1861,2±521,7***	***1332,3±342,8***	2980,6±952,8	2360,8±986,5	2036,6±535,7	1131,3±194,1
	**12:00-02:59 pm**	1760,3±386,1	1479,3±528,9	2775,9±479,6	2339,4±1024,1	2131,6±531	1494,6±152,4
	**03:00-05:59 pm**	2150,3±247,4	1901,7±694,6	***1992,7±427,8***	2608,9±1502,8	***1915,3±263,3***	***929,9±104,6***
**Night**	**06:00-08:59 pm**	2936,5±471,9	2660,6±623,2	***3314,7±606,3***	***3211,3±1066,8***	2610,2±626,7	1653±222,3
	**09:00-11:59 pm**	***3516,3±300,7***	***3054±900,5***	2957,6±513	2960,8±1125,7	***3079,5±556,8***	1688,6±334,4
	**12:00-02:59 am**	2817,2±558,4	1907,8±243,9	2700,1±479	***1662,1±598,8***	2654±485,2	1550±261,8
	**03:00-05:50 am**	2110,3±234,2	1557,5±493,9	2929,1±901,5	2068,8±636,5	2013,4±375,4	***1711,2±239,9***

LFD, low-fat diet. HFD, high-fat diet.

Supplemental Table S24: Overview of diet composition.

Diet	Chow diet	Low-fat/ low-sugar diet	High-fat/ low-sugar diet
**Catalog number (Envigo)**	**TD.2918**	**TD.180127**	**TD.06414**
**Kcal/g**	3.1	3.6	5.1
**Casein**	NR	210 g/kg	265 g/kg
**L-Cysteine**	0.3 %	3 g/kg	4 g/kg
**Corn Starch**	NR	465 g/kg	-
**Maltotextrin**	-	100 g/kg	160 g/kg
**Sucrose**	-	90 g/kg	90 g/kg
**Lard**	-	20 g/kg	310 g/kg
**Soybean Oil**	-	20 g/kg	30 g/kg
**Cellulose**	14.7 % [a]	37.15 g/kg	65.5 g/kg
**Calcium Phosphate, dibasic**	1.0 %	2 g/kg	3.4 g/kg
**Minerals**	added	35 g/kg [b]	48 g/kg [b]
**Fatty acids**	added	NR	NR
**Vitamins**	added	15 g/kg [c]	21 g/kg [c]
**Cholesterol**	-	-	-
**Choline Bitartrate**	1.2 g/kg	2.75 g/kg	3 g/kg
**Food color**	-	0.1 g/kg, orange	0.1 g/kg, blue
**Crude Fiber**	3.5 %	-	-
**Ash**	5.3 %	-	-
**Protein**	24.0 % kcal	20.5% kcal	18.3% kcal
**Fat**	18.0 % kcal	10.5 % kcal	60.3% kcal
**Carbohydrate**	58.0 % kcal	69.1 % kcal	21.4% kcal

NR, not reported; a, Estimate for ‘neutral detergent fiber’ which includes cellulose, hemicellulose, and lignin; b, Mineral Mix, AIN-93G-MX (94046); c, Vitamin Mix, AIN-93-VX (94047).
